# Supplementary material for: Phylogenetic Relationships of Turkish Indigenous Donkey Populations Determined by Mitochondrial DNA D-loop Region
Source: Animals (Basel). 2020 Oct 27;10(11):1970. doi: 10.3390/ani10111970 (PMC7692571; doi:10.3390/ani10111970)
Supplement: Supplementary file 1 [file animals-10-01970-s001.zip › Phylogenetic relationships of Turkish Native Donkey Populations determined by mitochondrial DNA D -loop region.docx]

Phylogenetic relationships of Turkish Native Donkey Populations determined by mitochondrial DNA D -loop region

Emel Özkan Ünal*^1^, Fulya Özdil*^2^, Selçuk Kaplan^3^, Eser Kemal Gürcan^1^, Serdar Genç^4^, Sezen Arat^2^ and Mehmet İhsan Soysal^1^

^1^ Tekirdağ Namık Kemal University, Faculty of Agriculture, Department of Animal Science, Tekirdağ/Turkey; ozemel@nku.edu.tr (E.Ö.Ü.); egurcan@nku.edu.tr (E.K.G.); misoysal@nku.edu.tr (M.I.S)

^2^ Tekirdağ Namık Kemal University, Faculty of Agriculture, Department of Agricultural Biotechnology, Tekirdağ/Turkey; fozdil@nku.edu.tr (F.Ö.); sarat@nku.edu.tr (S.A.)

^3^ Tekirdağ Namık Kemal University, Faculty of Veterinary Medicine, Tekirdağ/Turkey; [skaplan@nku.edu.tr](mailto:skaplan@nku.edu.tr)

^4^ Kırşehir Ahi Evran University, Department of Agricultural Biotechnology, Kırşehir /Turkey; serdargenc1983@gmail.com

***** Correspondence: ozemel@nku.edu.tr; Tel.: +90-282-250-2185 (E.Ö.Ü.)

fozdil@nku.edu.tr Tel: +90-282-250-2233 (F.Ö.);

Received: date; Accepted: date; Published: date

**Simple Summary:** This study presents the first substantial analysis of mtDNA diversity in Turkish native donkeys and provides information about the origin and genetic structure of donkey populations dispersed to 7 geographical regions in Turkey, and thus reveals insights into their genetic history. The phylogenetic tree and median-joining network exhibit two different maternal origins of the 16 Turkish native donkey populations.

**Abstract:** In this study, to investigate the mtDNA D-loop region and the origin of maternal lineages of 16 different donkey populations and to assess the domestication of Turkish native donkeys in 7 geographical regions, we analyzed the DNA sequences (383 bp of the D-loop region) of 315 native donkeys from Turkey. A total of 54 haplotypes resulting from 35 polymorphic sites (27 parsimoniously informative and 6 singleton sites) were defined, 28 of which (51.85 %) are unique and 26 are shared among different Turkish native donkey populations. The maximum composite likelihood estimates of the nucleotide substitution pattern, identified for the 315 sequences, were 33.00% (A), 27.3% (T), 28.6% (C), and 11.1% (G). The most frequent haplotype is H1 (45.71 %), followed by two haplotypes (H4 -15.55 % and H7-5.39 %), each with more than 10 samples. The breed genetic diversity evaluated by the haplotype diversity (H_D_) and nucleotide diversity (π_D_) for the Turkish donkey populations ranged from 0.533± 0.180 (MAL) - 0.933± 0.122 (AYD) and 0.01196± 0.0026 (ANT) - 0.02101 ± 0.0041 (AYD) respectively. We observed moderate-to-high levels of haplotype diversity and moderate nucleotide diversity indicating plentiful genetic diversity in all of the Turkish native donkey populations. Phylogenetic analysis (NJT) and Median-joining network analysis demonstrated that all haplotypes were clearly clustered into two major haplogroups—Clade I and Clade II, and each major clade contained several small sub-haplogroups. No congruence of haplogroup to a population’s geographic origin was observed. The results of AMOVA analyses based on geographic structuring of Turkish donkey populations highlighted that most of the observed variance is due to differences among samples within populations. The observed differences between groups were found to be statistically significant. Comparison among Turkish native donkey haplotypes and mtDNA D-loop sequences belonging to different country donkey breeds and wild asses, clearly define two clades referred to Somali and Nubian lineages. The results can be used to understand the origin of Turkish donkey populations clearly and to resolve the phylogenetic relationship among all of the different regions.

**Keywords:** Turkish donkeys; mtDNA; D-loop, genetic diversity, haplotype, maternal origin

1. Introduction

Animal domestication has played an important role in the appearance of modern civilizations. The identification of origin and phylogenetic relationships between animals is key for understanding of cultural changes in these civilizations and consequently has public and scientific attractions [1, 2]. The genetic origin was identified using three different genetic sources; nuclear genome, Y chromosome and mitochondrial genome [3]**.** Mitochondrial DNA (mtDNA) has been widely used in species identification and genetic diversification of domestic animals because of its special characteristics such as; more copies than nuclear DNA, well known gene structure, lack of introns, high mutation rate, absence of recombination events and maternal inheritance [4–6]. Moreover, very often it is the only genetic material that can be obtained from degraded or archaeological biological material. mtDNA has been used to investigate the history of modern domestic animals, i.e. to identify wild ancestors and to define domestication centers [7]. It is an important tool for phylogenetic studies and has been widely used to study the genetic differentiation, genetic complexity, evolutionary relationships, and origins of many domestic animals [8,9]. Including coding and non-coding regions, its length ranges from 15-20 kb in different species. The non-coding region is the control region (CR), also called the displacement-loop region (D-loop). mtDNA D-loop region is known to be more variable than other mitochondrial regions, and it is often used to analyze the phylogeny of closely related breeds within species [10]. It has a higher mutation rate than the cytochrome b (*Cyt-b)* gene. The length of the control region of the mtDNA of the donkey, excluding the repetitive portion, is 959 nt, one less than that of the horse. There are 108 differences (11.2%) in an alignment of the two regions, 88 transitions, 16 transversions, and four indels. The repetitive motifs occur in the same region in both species. The length of the repeated motif of the horse, 5’-GTGCACCT, is the same as that of the two different donkey motifs, but the sequence of each of the three motifs is highly distinct [11]. For this reason, these regions have been extensively used to investigate the maternal origin and genetic diversification of domestic donkeys [12,13]. Donkeys are one of the least studied large domestic animals, even though they are economically important in many regions of the world.

The process of domestication and dispersal route of the donkey (Equus asinus) is particularly interesting because the donkey has been widely used by humans to transport people and goods and till the land since approximately 5000 – 7000 years ago [14,15]. The domestication centers and the lineages of the donkey is still debated but the broadly accepted hypothesis, based on archaeological, historical and ethnographical sources data proposes that the domestic donkey has its origin mainly in the Nubian (*Equus africanus africanus*) and Somalian (*E. a. somaliensis*) ass. Analyses carried out on modern domestic donkey identified two mitochondrial haplogroups lineages, Clade 1 and Clade 2, and thus, provided evidence for two independent domestication events of donkeys [16–18]. Clade 1 is thought to derive from the Nubian wild ass (*E. a. africanus*) [16,18], whereas Kimura et al. [18] and Kefena et al. [19] recently found that Clade 2 donkeys may be descendants of a yet unrecognized extinct wild population rather than Somali wild ass. Beja -Pereira et al. [16] described high levels of genetic diversity in both the lineages of Northeast Africa territories and suggested that this area is one of the primary centers of donkey domestication. However, Kimura et al. [18] reported that the mtDNA sequences obtained from the extant Somali wild ass were classified together with the previously identified Somali wild ass specimens but failed to show any sequence similarity with domestic donkeys of both clades. This study suggested the existence of another ancestor of the domestic donkeys of clade 2, belonging to an additional yet unrecognized extinct wild population in Northeast Africa territories. In another study, based on genetic and on linguistic and zooarchaeological evidences it is confirmed that Ethiopia could be one of the major hotspots of donkey diversity and domestication [19].

Donkeys are an ancient component of Turkey’s community of domestic animals and have been important in the country’s agricultural economy for hundreds of years. In common with most of the world in the 21st century, however, the donkey in Turkey has lost its status as a mainstay of the rural economy and has been replaced by mechanical means of transport and power [20]. In ancient times, the donkey played a critical role in agriculture and transportation, especially in the rural areas of Turkey. Over the last 30 years, as farm mechanization and traffic automation have become more common, the number of donkeys has decreased sharply in the world [14].

In many developed countries the advent of the “conservation” strategies has resulted in many native breeds of many domestic species being described genetically and morphologically. In parts of Asian, European, and African donkeys have been described in mtDNA variation and genetic diversity. Turkey has a well-established and successful programme of classification, characterization and conservation of its major food producing domestic animals [21] and although the horse is included in this programme the donkey is not nor is covered by any related activities.

The goal of this study was: (i) to determine the genetic variability between seven different geographical regions donkey populations of Turkey (ii) to investigate the genetic structure of Turkish native donkey populations and to explore the maternal origin by means of mtDNA D-loop sequences, and domestic history of Turkish native donkey populations, (iii) to evaluate the presence of genetic breed sub-structuring and (iv) to compare mtDNA of Turkish populations with other countries domestic donkey breeds and wild asses. The results will be used to better understand the Turkish donkey population origins and to resolve the phylogenetic relationship among all the different regions.

2. Materials and Methods

*2.1. Population identification, sample collection and DNA isolation*

In this study a total of 315 blood samples were collected from Turkish native donkeys raised in 7 different geographical regions from 16 different provinces of Turkey (Figure 1). The provinces and the potential geographical distribution of these breeds with the number of sample sizes are given in Table 1. These provinces were known for their relatively large donkey populations in Turkey.


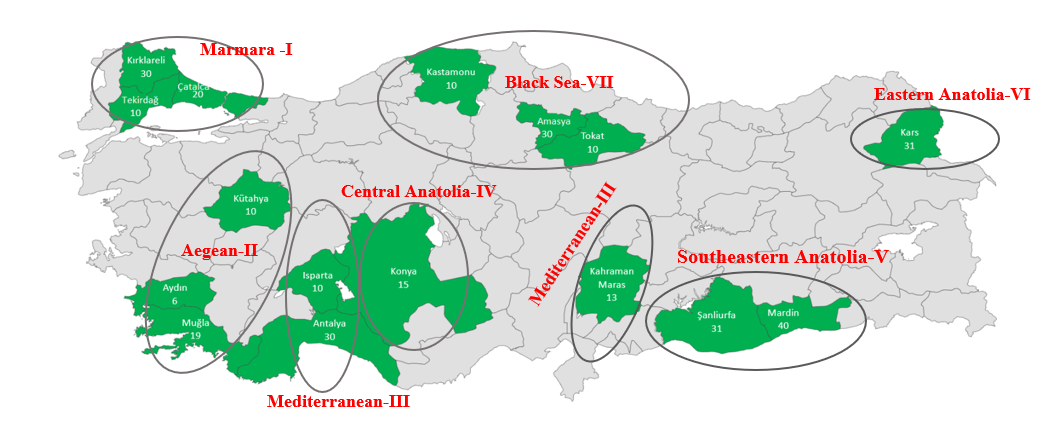


**Figure 1.** Turkish donkey populations sampling areas from the 7 different geographical regions of Turkey. The details of the regions are given in Table 1.

From those donkey individuals, eight to 10 mL of whole blood was collected from the jugular vein in EDTA-coated Vacutainer tubes (BD Vacutainer Systems, Plymouth, UK) and transported to the laboratory at 0 to 5 °C. Genomic DNA was extracted by using a standard phenol-chloroform extraction method [22]. The concentration of DNA was judged in comparison with the standard DNA marker concentration on agarose gels. The quality of DNA was checked on 0.6% agarose gels prepared with a Tris-Boric acid-EDTA buffer. Extracted DNA was diluted in TE buffer (10:1) and stored +4 ^0^C till analysis.

**Table 1** The regions, locations, the geographical locations with the number of sample sizes

| **Region** | **Locations** | **Geographical**  **Location** | **Number of samples** |
| --- | --- | --- | --- |
| **Marmara ( I )**  **(MRM)** | Kırklareli (KIR) | 41^o^51'N 27^o^19'E | 30 |
|  | İstanbul-Çatalca (CAT) | 41^o^06'N 28^o^30'E | 20 |
|  | Tekirdağ-Malkara (MAL)) | 40^o^52'N 26^o^57'E | 10 |
| **Black Sea ( VII )**  **(BSR)** | Amasya-Merzifon (MER) | 40^o^53'N 35^o^32'E | 30 |
|  | Tokat (TOK) | 40^o^12'N 36^o^27'E | 10 |
|  | Kastamonu-Cide (KAS) | 41^o^50'N 32^o^54'E | 10 |
| **Aegean ( II )**  **(AER)** | Kütahya (KUT) | 39^o^21'N 30^o^01'E | 10 |
|  | Muğla (MUG) | 36^o^37'N 29^o^26'E | 19 |
|  | Aydın (AYD) | 37^o^44'N 28^o^01'E | 6 |
| **Central Anatolia ( IV )**  **(CAR)** | Konya (KON) | 37^o^38'N 32^o^26'E | 15 |
| **Mediterranean ( III )**  **(MDR)** | Isparta (ISP) | 37^o^49'N 30^o^44'E | 10 |
|  | Kahramanmaraş (KRM) | 37^o^30'N 36^o^57'E | 13 |
|  | Antalya (ANT) | 36^o^50'N 30^o^13'E | 30 |
| **Eastern Anatolia ( VI )**  **(EAR)** | Kars (KAR) | 40^o^36'N 43^o^07'E | 31 |
| **South East Anatolia ( V )**  **(SAR)** | Mardin (MRD) | 37^o^18'N 40^o^44'E | 40 |
|  | Şanlıurfa (SAN) | 37^o^10'N 38^o^50'E | 31 |
|  | | **Total** | **315** |

*2.2. Amplification of mtDNA regions (D-loop) and sequencing*

The 383 bp segment from the mitochondrial DNA D-loop region from donkey genome was amplified by Polymerase Chain Reaction (PCR) as described by Aranguren-Méndez et al. [4]. The primer sequences amplified between the sites 15387 to 15769 bp. The PCR conditions were standardized for all the primer pairs selected for the study. D-loop gene region was amplified by PCR carried out in 20 µl reaction volume containing 50 ng of genomic DNA, 2.0 mM MgCl_2_, 0.2 mM of each dNTP, 0.5 µM of each primer, 1 X PCR Buffer and 1 U of Taq DNA polymerase (Fermentas, Thermo Fischer Scientific). Amplification was carried out using a BIORAD MyCycler Thermal cycler (BIORAD, USA) with the following conditions: initial denaturation at 94 ^0^C for 5 min followed by 35 cycles of denaturation at 94 ^0^C for 30 sec, annealing at 58 ^0^C for 30 sec extension at 72 ^0^C for 2 minutes and final extension at 72 ^0^C for 15 minutes. The amplified products were electrophoresed, visualized under UV radiations, and purified to be sequenced using ABI prism 3500 genetic analyzer (Applied Biosystems, Inc., Foster City, CA). PCR products were sequenced for both directions. The raw sequence trace files were checked for the presence of ambiguous bases using software Chromas v. 1.7.4 (http:// [www.technelysium.com.au/](http://www.technelysium.com.au/)).

*2.3. Data analyses*

Sequences were checked for the presence of ambiguous bases by using the software ChromasPro v.1.7.4 (<http://www.technelysium.com.au/>). In order to define the haplotypes (H-) all obtained sequences were aligned with the complete donkey mtDNA sequences MK896308 [23] and X97337 [11], widely used as reference sequences, using the ClustalW algorithm implemented in BioEdit v7.0.9 [24] and MEGA v.7.0.26 software [25]. The number of haplotypes (N_H_), number of private haplotypes (P_H_), haplotype diversity (H_D_), total polymorphic sites (S), parsimony informative sites (SPI), singleton site (S_S_), shared haplotype (S_H_), the nucleotide diversity (**π_D_**) and average number of nucleotide differences (k) were estimated using the DNASP 6.12.03 x64 software [26]. Pairwise population genetic differentiation (F_ST_) and the average number of pairwise differences within and between populations were calculated using Arlequin v.3.5.2.2 [27]. In order to define the genetic relationships among Turkish native donkey populations mtDNA sequences and those available in Genbank for other donkey populations originated from the European, Asian and African origin domestic donkeys, Ethiopian donkeys and wild asses, a total of 429 sequences were considered. All the sequences were trimmed at 344 bp to maximize the number of sequences included. The mtDNA sequences from GenBank database used for haplogroup definition and relationship investigation were reported in Table S1. To assess the genetic variance among donkeys within and between populations, we performed the analysis of molecular variance (AMOVA) using Arlequin v.3.5.2.2 [27]. For the neighbor-joining tree reconstruction and the median-joining network (MJN), we used MEGA v.7.0.26 software [25], T-REX web server and NETWORK 10.1.0.0 software [28], respectively. All mtDNA D-loop sequences determined in this study were deposited in the GenBank with the accession numbers MH683672 - MH683725.

3. Results

3.1. Genetic diversity of the D-loop region analyses of Turkish donkey population

The mtDNA D-loop sequences were obtained for all analyzed 315 samples from 7 different regions from 16 different provinces of Turkey. The haplotype and nucleotide diversities of the sampled populations were presented in Table 2. The sequencing results of mtDNA D-loop of Turkish native donkey populations revealed 54 different haplotypes (Figure 2.a) which were designated H_1_ to H_54_, (GenBank accession nos. MH683672-MH683725) resulting from 35 polymorphic sites (27 parsimoniously informative and 6 singleton sites). The table with haplotype Turkish native donkeys is presented in Supplementary Materials Table 1. The haplotype distribution by clusters corresponding to the Turkish native donkeys is shown in Figure 2.b. Construction of the phylogenetic tree by the maximum likelihood method showed that two different cluster. The maximum composite likelihood estimates of the nucleotide substitution pattern, identified for the 315 sequences, were 33.00% (A), 27.3% (T), 28.6% (C), and 11.1% (G). Positions containing gaps and missing data were eliminated. Of the 54 haplotypes, 28 are unique and 26 are shared among different Turkish native donkey populations. The most frequent haplotype is H1 (45.71 %), with 144 samples from 16 different populations, followed by two haplotypes (H4 -15.55 % and H7-5.39 %), each with more than 10 samples. H1, determined in 45.71% of donkeys (n = 144), had the highest frequency in the studied sample, while in the SAR region donkey population, its frequency was 23.61% (n = 34), in MRM, 22.91% (n = 33), in MDR, 18.75% (n=27); in BSR, 15.27% (n= 22), in EAR,12.5% (n=18), in AER 5.56% (n=8) and in CAR, 1.38% (n = 2). High frequency (15.55%, n = 49) and representation in seven region populations (SAR, 32.65%, n = 16; AER, 18.36%, n = 9; MRM and CAR, 12.24%, n = 6; in MDR, 10.20%, n=5; in EAR, 8.16%, n=4; in BSR, 6.12%, n=3) was deﬁned for H4. In total, only three haplotypes were present in all groups—these are the abovementioned H1, H4 and H7.

The observed haplotypes, their annotation and frequencies in studied populations are presented in Table 3. The 26 haplotypes were shared between populations (H_1_, H_2_, H_4_, H_5_, H_6_, H_7_, H_8_, H_9_, H_10_, H_11_, H_12_, H_13_, H_14_, H_15_, H_16_, H_17_, H_20_, H_23_, H_25_, H_26_, H_27_,H_29_, H_32_, H_33_, H_34_, H_37_), whereas the remaining 28 were unique. Haplogroup H_1_, which is thought to be the oldest haplogroup. The regions with the highest number of haplotypes were found in Southeast Anatolia (21), Marmara (19), Mediterranean (18) and Black Sea (17) regions. Southeast Anatolia populations have higher mtDNA diversity with 21 haplotypes. 11 of these haplotypes were observed only in Southeast Anatolia populations. It has been observed that the lowest haplotype diversity is in Central Anatolia and Eastern Anatolia. It is thought that the reason of this decrease will be due to the low number of individuals and the sampling of only one province in these regions. The haplotype number in each population varies from 4 to 15. The highest haplotype number was observed in (MRD) donkeys followed by (MER), (KIR) and (ANT) donkey populations. The lowest haplotype number was observed in (MAL, KUT, KRM) donkeys followed by (TOK) and (AYD) donkey populations.

The breed genetic diversity (Table 2) evaluated by the haplotype diversity (H_D_) and nucleotide diversity (π_D_) for the Turkish donkey populations ranged from 0.533± 0.180 (MAL) - 0.933± 0.122 (AYD) and 0.01196± 0.0026 (ANT) - 0.02101 ± 0.0041 (AYD) respectively. The overall values for haplotype and nucleotide diversities were 0.763±0.023 and 0.01656±0.00046 respectively (Table.1). Analysis of mtDNA D-loop region for each population revealed that (AYD) donkey population had the highest haplotype diversity followed by (KAS) donkey population. The lowest haplotype diversity was observed in (MAL) donkeys followed by (KAR) and (SAN) donkey populations. (AYD) donkeys had the highest estimates of nucleotide diversity followed by (ISP) donkey population. Generally, estimate of nucleotide diversities ranged from 0.01196 in (ANT) donkeys to 0.02101 in (AYD) donkeys. The (KRM) donkey population had the highest number of private haplotypes (n=8). In addition, the average number of nucleotide differences (k) ranged from 4.172 (ANT) to 7.333 (AYD) with an average value of 5.745.

| **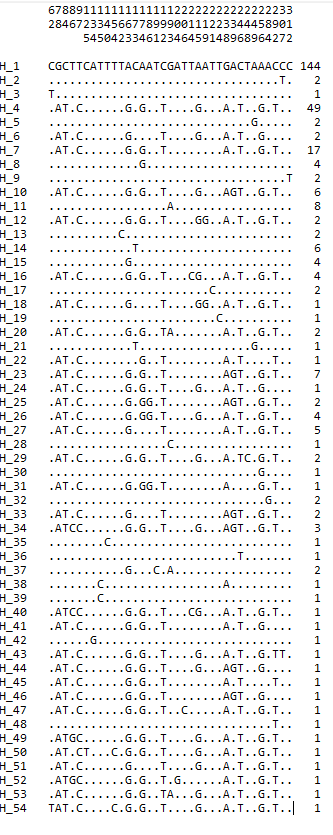(a)** | **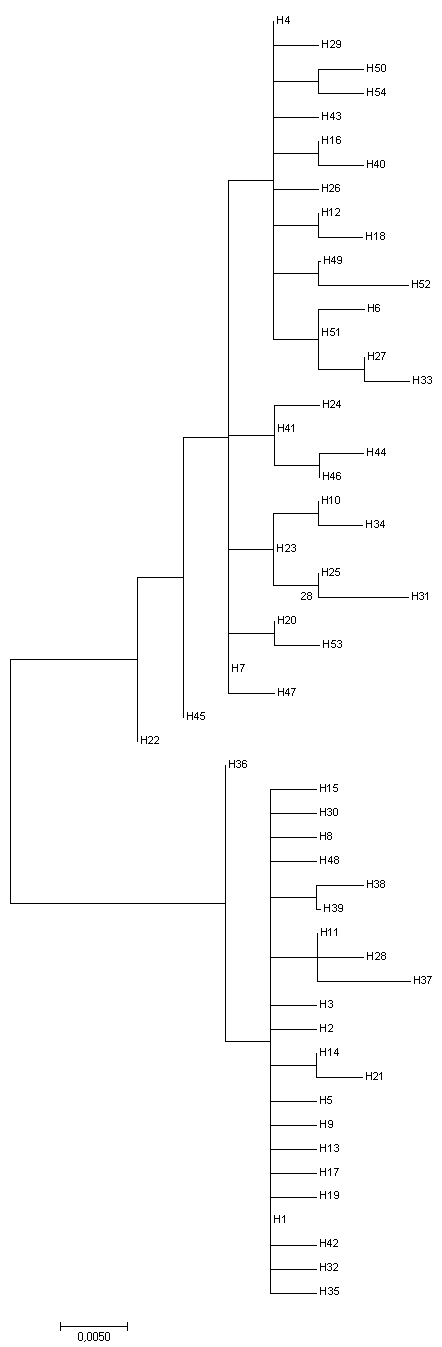(b)**  II  **I** |
| --- | --- |

**Figure 2. a)** Polymorphic sites and haplotypes **b)** Molecular phylogenetic analysis by Maximum Likelihood method distribution of haplotypes in Turkish native donkeys.

**Table 2.** D-loop nucleotide polymorphisms and molecular diversity indices of the 16 Turkish donkey populations.

| **Population** | **n** | **N_H_** | **H_D_± SD** | **S** | **SPI** | **S_S_** | **P_H_** | **S_H_** | **π_D_ ± SD** | **k** | **Region** |
| --- | --- | --- | --- | --- | --- | --- | --- | --- | --- | --- | --- |
| **KIR** | 30 | 12 | 0.710±0.007 | 18 | 11 | 7 | 1 | 11 | 0.01393±0.0025 | 4.848 |  |
| **CAT** | 20 | 11 | 0.763±0.0106 | 19 | 11 | 8 | 2 | 9 | 0.01288±0.0036 | 4.495 | MRM |
| **MAL** | 10 | 4 | 0.533±0.180 | 12 | 11 | 1 | - | 4 | 0.01496±0.0044 | 5.222 |  |
| **MER** | 30 | 13 | 0.720±0.091 | 16 | 15 | 1 | 3 | 10 | 0.01446±0.0023 | 5.048 |  |
| **TOK** | 10 | 5 | 0.756±0.130 | 13 | 12 | 1 | - | 5 | 0.01821±0.0032 | 6.356 | BSR |
| **KAS** | 10 | 7 | 0.911±0.077 | 14 | 12 | 2 | 2 | 5 | 0.01731±0.0036 | 6.022 |  |
| **KUT** | 10 | 4 | 0.778±0.091 | 13 | 12 | 1 | - | 4 | 0.01681±0.0038 | 5.867 |  |
| **MUG** | 19 | 9 | 0.871±0.048 | 16 | 11 | 5 | 2 | 7 | 0.01696±0.0020 | 5.918 | AER |
| **AYD** | 6 | 5 | 0.933±0.122 | 14 | 10 | 4 | - | 5 | 0.02101±0.0041 | 7.333 |  |
| **KRM** | 13 | 4 | 0.654±0.106 | 12 | 11 | 1 | - | 4 | 0.01646±0.0026 | 5.744 |  |
| **ISP** | 10 | 7 | 0.867±0.107 | 15 | 12 | 3 | 1 | 6 | 0.01917±0.0033 | 6.689 | MDR |
| **ANT** | 30 | 12 | 0.717±0.090 | 18 | 15 | 3 | 5 | 7 | 0.01196±0.0026 | 4.172 |  |
| **KON** | 15 | 7 | 0.829±0.082 | 14 | 12 | 2 | 3 | 4 | 0.01659±0.0027 | 5.791 | CAR |
| **KAR** | 31 | 9 | 0.652±0.091 | 15 | 12 | 3 | 1 | 8 | 0.01455±0.0023 | 5.079 | EAR |
| **MRD** | 40 | 15 | 0.758±0.060 | 18 | 12 | 6 | 8 | 7 | 0.01753±0.0008 | 6.118 | SAR |
| **SAN** | 31 | 9 | 0.695±0.076 | 17 | 13 | 4 | 3 | 6 | 0.01764±0.0012 | 6.155 |  |
|  | 315 | 54 | 0.763±0.023 | 35 | 29 | 6 |  |  | 0.01680±0.00046 | 5.745 |  |

Sample size (n), number of haplotypes (N_H_), haplotype diversity (HD) with their standard deviation (SD), total polymorphic sites (S), parsimony informative sites (SPI), singleton site (S_S_), private haplotype(P_H_), Shared Haplotype (S_H_), nucleotide diversity (**π_D_**) with their standard deviation (SD) and average number of nucleotide differences (k) within and across the 16 populations.

**Table 3.** The number of haplotypes observed by regions.

| **Region** | **N_H_** |  |  |  |  |  |  |
| --- | --- | --- | --- | --- | --- | --- | --- |
| **MRM** | 19 | H_1_, H_2_, H_3_, H_4_, H_5_, H_6_, H_7_, H_8_, H_9_, H_10_, H_11_, H_12_, H_13_, H_14_, H_15_, H_16_, H_17_, H_18_, H_19_ | | | | | |
| **BSR** | 17 | H_1_, H_4_, H_5_, H_7_, H_8_, H_11_, H_14_, H_20_, H_21_, H_22_, H_23_, H_24_, H_25_, H_26_, H_27_, H_28_, H_29_ | | | | | |
| **AER** | 12 | H_1_, H_4_, H_7_, H_8_, H_13_, H_14_, H_16_, H_23_, H_25_, H_34_, H_41_, H_42_ | | | | | |
| **MDR** | 18 | H_1_, H_2_, H_4_, H_6_, H_7_, H_11_, H_15_, H_16_, H_23_, H_27_, H_33_, H_34_, H_35_, H_36_, H_37_, H_38_, H_39_, H_40_ | | | | | |
| **CAR** | 7 | H_1_, H_4_, H_7_, H_12_, H_30_, H_31_, H_32_ | | | | | |
| **EAR** | 9 | H_1_, H_4_, H_7_, H_8_, H_10_, H_11_, H_14_, H_15_, H_43_ | | | | | |
| **SAR** | 21 | H_1_, H_4_, H_7_, H_10_, H_11_, H_14_, H_17_, H_20_, H_26_, H_34_, H_44_, H_45_, H_46_, H_47_, H_48_, H_49_, H_50_, H_51_, H_52_, H_53_, H_54_ | | | | | |

The number of haplotypes (N_H_)

The 54 haplotypes were defined by 29 parsimony informative sites. The parsimony informative sites [25] are defined as mutations that have a minimum of two nucleotides that are present at least twice in the sampled population, whereas noninformative sites are singleton sites. Of the polymorphic sites, there were 35 transitions and 2 transversions, and two sites had both a transition and transversion, suggesting a strong bias toward transitions. No insertions or deletions were observed. At population level, all the breeds showed one or more singleton sites. As reported in Table 2 the (CAT) population counted the highest number of non-informative singleton sites (S_S_), as well as the lowest number of polymorphic sites (S) was identified in (MAL) and (KRM) populations. In addition, the average number of nucleotide differences (k) was the highest for (AYD) donkeys (7.333) followed by (ISP) donkeys (6.689) while (ANT) donkeys had the lowest average number of nucleotide differences (4.172). The AYD breed showed five haplotypes, the highest **π** value (0.02101), the highest number of haplotype diversity (0.933) and the highest nucleotide diversity (k=7.333). The high k value for the AYD breed is due to the high difference among the five haplotypes identified in the population. Despite the low number of samples, a high H_D_ and (π) value, with five haplotypes (H1, H4, H7, H14 and H25) for the AYD was found (Table 1). On the contrary ANT have the lowest **π_D_** value (0.01196) and k value (k=4.172), but haplotypes have a high H_D_ value (0.717).

The matrix of pairwise F_ST_ (fixation index) value and D_A_ distances value between Turkish donkey populations from 7 geographical regions are shown in Table 4. The F_ST_ values range from -0.0302 (Aegean vs. Central Anatolia) to 0.2523 (Marmara vs. Central Anatolia). The highest differences in F_ST_ values were found between the Marmara and Central Anatolia region populations (F_ST_: 0.2523, P<0.01) followed by between Mediterranean and Central Anatolia region donkey populations (F_ST_: 0.2130, P<0.01). In contrast, there is little genetic differentiation between Southeastern Anatolia and Black Sea donkeys (F_ST_:0.0004) followed by between Mediterranean and Black Sea (F_ST_:0.0068). In the study, some F_ST_ values were observed to be negative (Table. 4). Negative F_ST_ values should be effectively seen as zero values. A zero value for F_ST_ means that there is no genetic subdivision between the populations considered.

**Table 4.** Matrix of pairwise F_ST_ (below diagonal) and D_A_ distances (D-loop nucleotides sequence divergence, above diagonal) between 16 Turkish native donkey populations.

|  | **MRM** | **BSR** | **AER** | **MDR** | **CAR** | **EAR** | **SAR** |
| --- | --- | --- | --- | --- | --- | --- | --- |
| **MRM** | - | 0.00036 | 0.00391 | -0.00014 | 0.00465 | -0.00024 | 0.00115 |
| **BSR** | 0.0242 | - | 0.00139 | 0.00011 | 0.00190 | -0.00007 | 0.00001 |
| **AER** | 0.2106^**^ | 0.0765^*^ | - | 0.00323 | -0.00049 | 0.00273 | 0.00052 |
| **MDR** | -0.0099 | 0.0068 | 0.1734^**^ | - | 0.00399 | -0.00026 | 0.00082 |
| **CAR** | 0.2523^**^ | 0.1027^*^ | -0.0302 | 0.2130^**^ | - | 0.00340 | 0.00069 |
| **EAR** | -0.0171 | -0.0052 | 0.01464^*^ | -0.0184 | 0.1843^**^ | - | 0.00047 |
| **SAR** | 0.0685^*^ | 0.0004 | 0.0291 | 0.04799^*^ | 0.0376 | 0.0263 | - |

^*^ P < 0.05; ^**^P <0.01

Also, the matrix of pairwise F_ST_ values between Turkish native donkey populations from 16 different provinces is shown in Figure 3. The F_ST_ values range from -0.1184 (TOK-MUG) to 0.3446 (KUT-KRM). Out of 120 pairwise F_ST_ values 28 comparisons had F_ST_ values between 0 and 0.05 showing little genetic differentiation while 16 comparisons had F_ST_ values between 0.05 and 0.15 showing moderate genetic differentiation, 13 comparisons had F_ST_ values between 0.15 and 0.25 showing great genetic differentiation, 8 comparisons had F_ST_ values greater than 0.25. Negative F_ST_ values were recorded in some comparisons and these equate to zero F_ST_ values (Supplementary Table S_2_). While most of the highest F_ST_ (> 0.25) values were seen between AER, MRM, MDR, BSR and CAR region populations – KUT-KIR, KUT-CAT, KUT-KRM, KRM-AYD, KRM-KAS, KAS- CAT, KON-CAT and KON-KRM. These populations showed values corresponding to very great genetic differentiation (Figure 3).


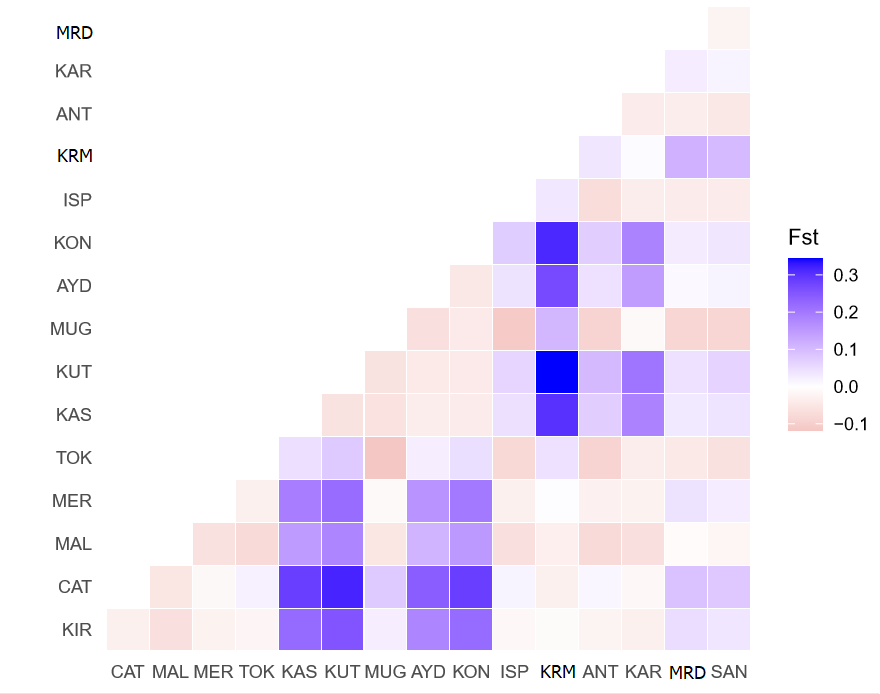


**Figure 3.** Matrix of pairwise fixation indexes (F_ST_) calculated for the 16 Turkish native donkey populations (F_ST_ values: Supplementary Table S2).

A Neighbor - joining tree (NJT) from Reynolds’ linearized genetic distances (Supplementary Table S3) for 315 tested sequences from different regions was constructed using the T-REX web server. NJT clearly demonstrates three distinct clades in Turkish donkey populations from 7 geographical regions (Figure 4). Three geographical regions, including CAR, AER, SAR, clustered in clade I, while different three geographical region populations, including MDR, MRM and EAR, clustered in clade II. Clade III, only one population (BSR) is included. The topology of this tree reflected the patterns of Reynolds's linearized pairwise genetic distances where MRM and MDR, MRM and EAR, BSR and EAR, CAR and AER, MDR and EAR as well as BSR and SAR region donkeys were genetically much closer to each other than to the rest of pairwise genetic distances.


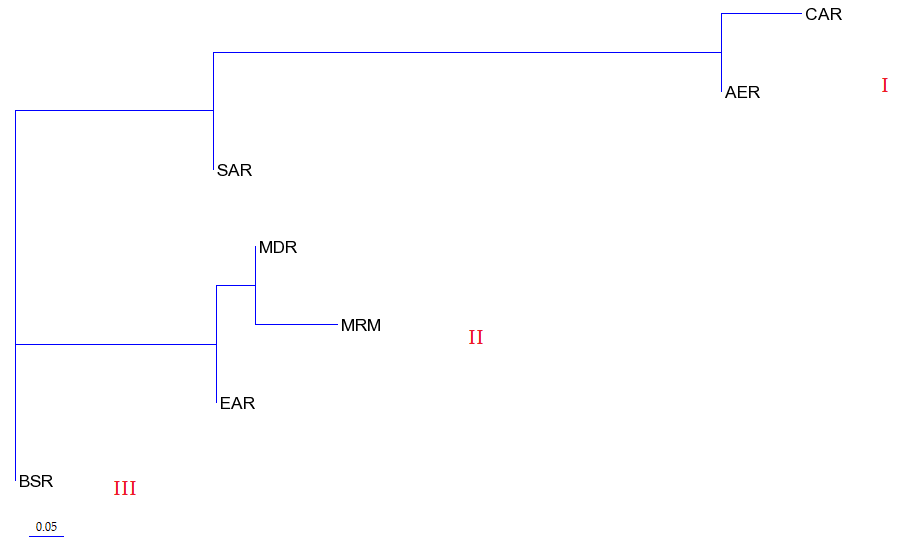


**Figure 4.** Neighbor-joining dendrogram constructed from Reynolds’ linearized genetic distances in Turkish donkey populations from different regions. (CA: Central Anatolia; AEG: Aegean; SEA: Southeastern Anatolia; MED: Mediterranean; MAR: Marmara; EA: Eastern Anatolia, BS: Black Sea)

The network reconstructed using median-joining (MJ) algorithm portrayed ancestral or unsampled sequences as median vectors (mv) and the frequency of haplotypes were represented by the size of the circle (Figure 5). Two major clades were obtained from the reconstructed network. Clade 1 lineage was obtained from 31 haplotypes (H4, H6, H7, H10, H12, H16, H18, H20, H22, H23, H24, H25, H26, H27, H29, H31, H33, H34, H40, H41, H43, H44, H45, H46, H47, H49, H50, H51, H52, H53, H54). This clade was derived mainly from haplotypes obtained from SAR (n=32), AER (n=22), BSR (n=20), MRM (n=15), MDR (n=15), CAR (n=10) and EAR (n=9) region donkey populations. Clade 2 lineage was formed from 23 haplotypes (H1, H2, H3, H5, H8, H9, H11, H13, H14, H15, H17, H19, H21, H28, H30, H32, H35, H36, H37, H38, H39, H42, H48) and it was separated from Clade 1 by 5 mutation steps (mutation steps shown Figure 5). This clade was dominated by haplotypes obtained from MRM (n=45), MDR (n=38), SAR (n=38), BSR (n=30), EAR (n=22), AER (n=13) and CAR (n=5) region donkey populations. Star-like clusters were seen around the haplotypes H1 and H4 in MJ tree and localization of the haplotypes showed the similar topology with NJ tree (Figure 4).

| 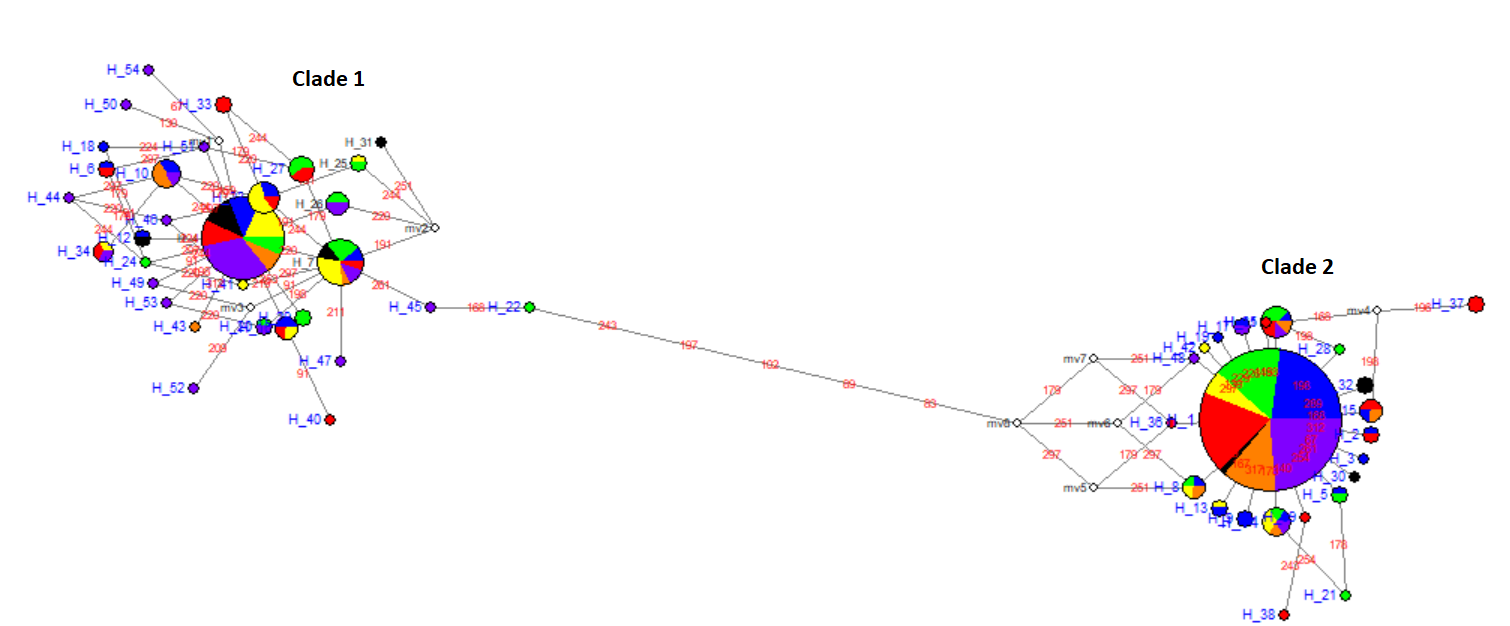 | |
| --- | --- |
| 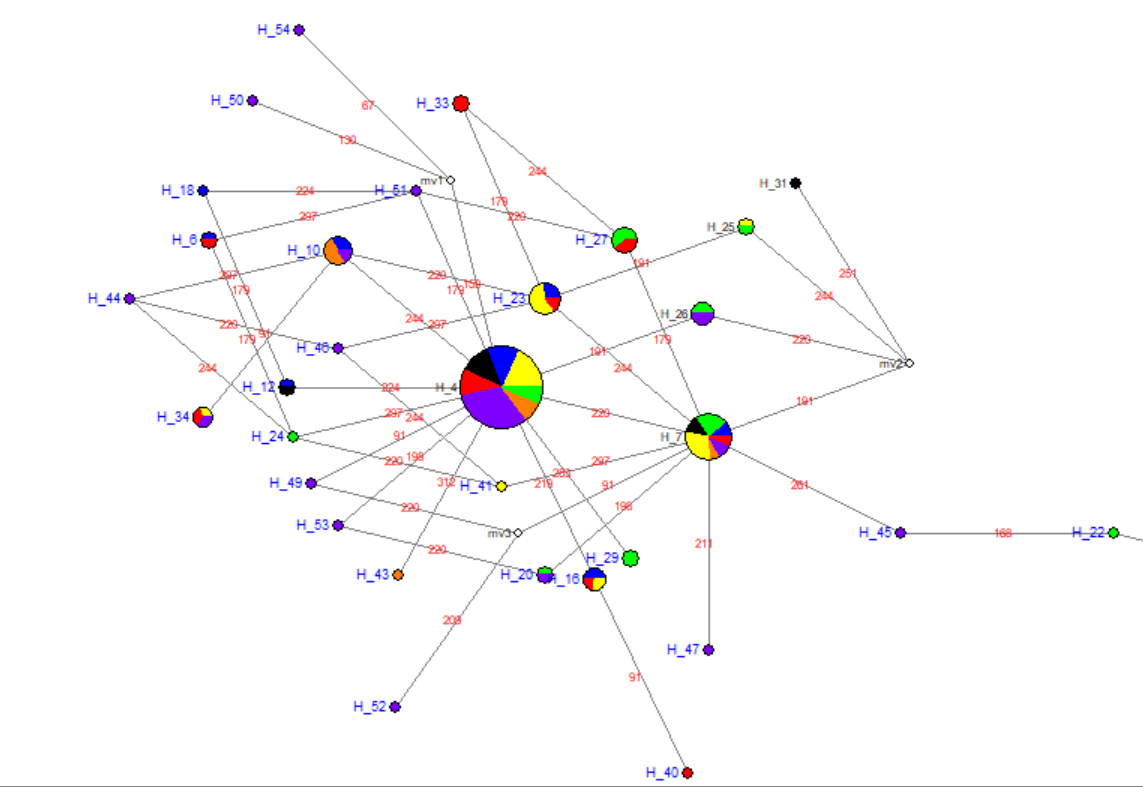Clade I | 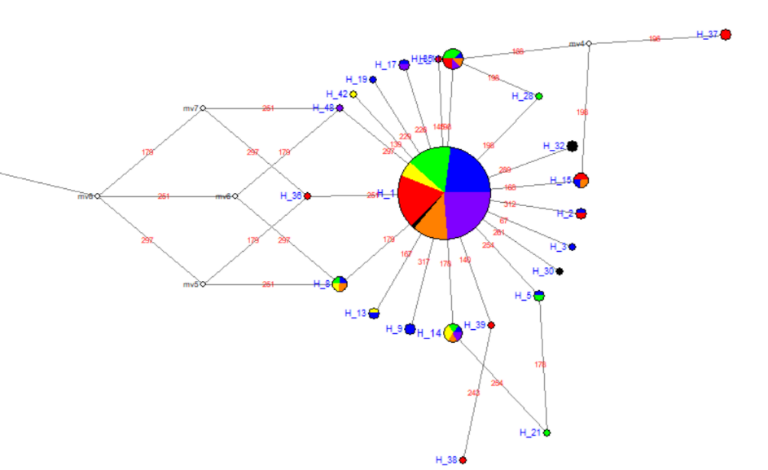  Clade II |

**Figure 5.** Median-joining network constructed from 54 haplotypes obtained from 7 different geographical regions and 16 different populations. Median vectors (mv) are denoted by white circles representing extant unsampled or extinct ancestral sequences. Circle areas are proportional to haplotype frequencies and the numbers of mutational steps are not shown. (MRM: Blue, BSR: Green, AER: Yellow, MDR: Red, CAR: Black, EAR: Orange, SAR: Purple)

*3.2. Turkish native donkey populations comparing with other donkey populations*

Comparison of the 427 reference (the GenBank database samples, supplementary Table S1) mtDNA D-loop sequences and Turkish native donkey populations sequences of 344 bp revealed 88 polymorphic sites (**Supplementary Figure 1**). Using all variations, 145 haplotypes were derived, differing from each other by one to 24 variations. Twenty-four haplotypes were newly discovered in Turkish native donkeys (H77-89, H92-93, H95-103). Twenty-five haplotypes were shared among the Turkish native donkeys we collected from different countries donkeys (H1-3, H5, H11-12, H14-15, H17-19, H21-22, H37, H44, H54-55, H57, H64-66, H68, H86, H90-91). While common 25 haplotypes were known from Turkish native donkey populations and other country donkeys, we identified two novel haplotypes in MRM region donkeys (CAT: H81, H82), four in BSR region donkeys (MER: H83, H84, H85; KAS:H87), two in CAR region donkeys (KON: H88, H89), two in MDR region donkeys (ANT: H92, H93), one in EAR region donkey (KAR: H95) and eight in SAR region donkeys (SAR:H96, H97, H98, H99, H100, H101, H102, H103) (supplementary Figure 1). One haplotype belong to the MRM and BSR region donkeys (H77: KIR, MER), 1 to MRM and MDR region donkeys (H78: KIR, ANT), 1 to MRM and AER region donkeys (H79: CAT, MUG) and one to MRM, MDR and EAR region donkeys (H80: CAT, ANT, KAR).

The haplotype diversity and nucleotide diversity of the mtDNA D-loop region in different country donkeys (427 reference mtDNA D-loop sequences) and Turkish native donkeys are shown in Table 5.

**Table 5.** Analysis of the haplotype and nucleotide diversity of mtDNA D-loop region within different donkey populations

| **Population** | **n** | **H_D_± SD** | **π_D_ ± SD** | **Referance** |
| --- | --- | --- | --- | --- |
| **Turkish native donkeys** | 315 | 0.763±0.023 | 0.01680± 0.00046 | This study |
| **Different country donkeys** | 10 | 0.956±0.059 | 0.02349±0.00598 | [11,29–33] |
| **African origin domestic donkeys** | 85 | 0.983±0.007 | 0.02843±0.00195 | [16] |
| **Chinese donkeys** | 146 | 0.890±0.013 | 0.01759± 0.00053 | [17] |
| **Balkan donkeys** | 62 | 0.973±0.011 | 0.01764±0.00067 | [34] |
| **Serbian donkeys** | 23 | 0.949± 0.033 | 0.01922±0.00136 | [15] |
| **Nigerian, Iranian, Tadzhikistan, Chinese, Kenya and Kyrgyzstan donkeys** | 73 | 0.965± 0.010 | 0.02613± 0.00374 | [23] |
| **Italian donkeys** | 28 | 0.913± 0.033 | 0.01568± 0.00133 | [35] |

The phylogenetic relationship among the 145 identified haplotypes is calculated through the Median-Joining Network. MJN revealed that there are five distinct lineages, as shown in the phylogenetic tree and the star-like phylogeny (Figure 6a). The haplotypes of each population are identified by the color code, the abundance by the relative size of the symbol and the diffusion among the breeds with the pie division of the various colors (Figure 6b, 6c).

| 1. 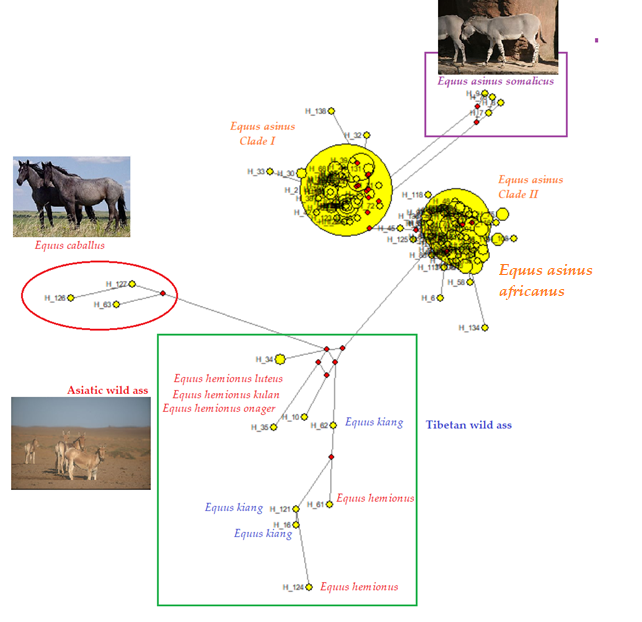   **V**  **IV**  **III**  **II**  **I** |
| --- |
| 1. IV lineage 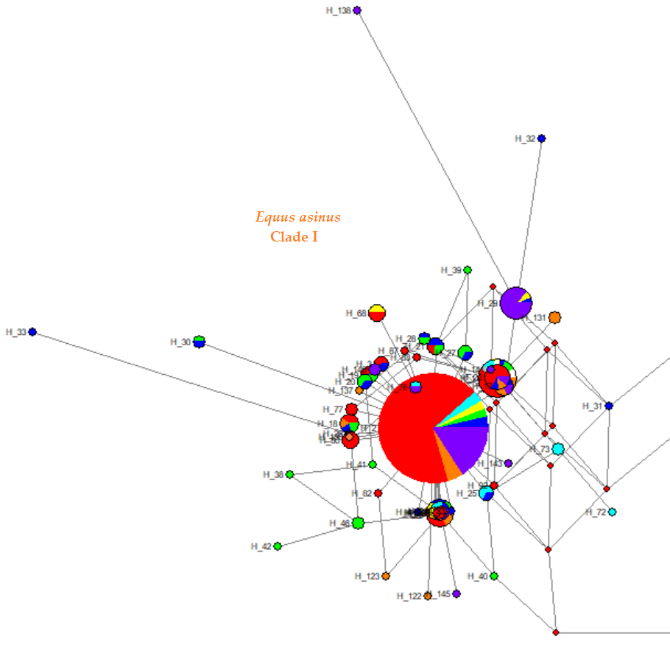 |
| **c)** V lineage 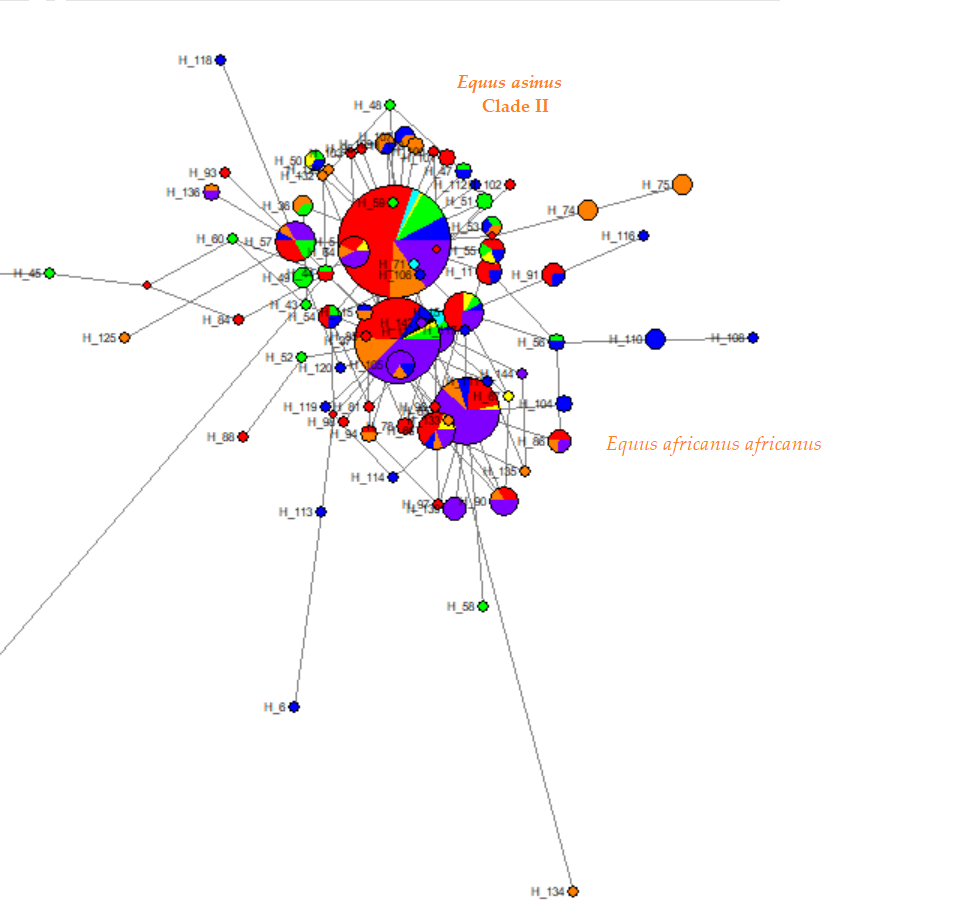 |
| **Figure 6.** a) MJN based on the 16 different region Turkish native donkey populations and the 429 donkey sequences from GenBank. Circle are proportional to the haplotypes frequencies; black circles are median vectors (mv) representing extant unsampled or extinct ancestral sequences; in red the number of mutation point respect to the reference sequence X97337. b) IV; Clade I: Somali lineages (*Equus asinus somalicus*), c) V; Clade II: Nubian lineage (*Equus africanus africanus*). (Red: Turkish native donkey populations; Blue: [16], Green: [34], Yellow: [15], Orange: [23], Purple: [17], White: [11], Turquoise: [35], Grey: [31]). |

The MJN analysis identified 145 haplotypes from 742 mtDNA D-loop sequences (427 GenBank sequences (Supplementary Table S1) and our 315 Turkish native donkeys) showing a high variability (H_D_=0.890±0.009). The reduced median network revealed that there are five distinct lineages, as shown in the phylogenetic tree and the star-like phylogeny. Three samples—H126, H127, and H63—were horses (*Equus cabullus*) that grouped together as outgroup to the donkeys in the phylogenetic tree. *Equus cabullus*, *Equus kiang* and Asiatic wild asses (*E. hemionus luteus, E. hemionus kulan, E. Hemionus onager*) shared (I) and (II) lineage and had their respective lineages. Lineage (I) (*Equus cabullus*) have 3 haplotypes, lineage (II) (*E. hemionus luteus, E. hemionus kulan, E. Hemionus onager, Equus kiang)* has 8 haplotypes. Four haplotypes were identified in *E. asinus somalicus* and were distributed into one lineage (III). Lineages IV and V consists of domestic donkey populations. In Figure 6; Lineages IV and V which has two different clades; are clearly defined in domestic donkey populations: Clade 1 includes the 50.68 % of sequences, whereas Clade 2 includes the 49.31 % of sequences in domestic donkey populations. Table 6 shows that genetic distances and nucleotide diversity of two clades.

**Table 6.** Genetic distance and nucleotide diversity of two clades

| **Population** | **Pairwise distance± SD** | **π_D_ ± SD** |
| --- | --- | --- |
| **Clade I lineage** | 1.4228 ± 0.8715 | 0.004136± 0.002802 |
| **Clade II lineage** | 2.1506 ± 1.1992 | 0.00525 ± 0.003857 |

The AMOVA analysis is a useful tool to check how the maternal genetic diversity is distributed within and among populations. We analyzed different possible structures by creating and comparing different population groups. We ran the analysis under two hypotheses: Hypothesis 1; the breeds have been clustered in five groups: (1) Turkey’s native donkey populations (n=315) (2) Balkan donkeys [34], Italian donkeys [35], Serbian donkeys [15] (n=112) (3) China, Tadzhikistan, Kyrgyzstan, Iran, Kenya, Mongolia donkeys (n=219) [17,23] (4) African origin domestic donkeys (n=85) [16] (5) wild asses and others (n=11) [11,29–33,36]*.* Hypothesis 2; three groups according to Neighbor-joining dendrogram constructed from Reynolds’ linearized genetic distances in Turkish donkey populations from different regions. (1) CAR, AER, and SAR region donkeys; (2) MDR, MRM and EAR region donkeys and (3) BSR region donkeys. Table 7 reports the results of the AMOVA analysis according to our hypotheses. Hypothesis 1; the AMOVA analyses results showed that the variation among groups, among populations within groups, and within populations were 7.95%, 1.72% and 90.34%, respectively. The results highlight that most of the observed variance is due to differences within populations. Variance components among groups were nonsignificant, demonstrating not significant geographical distribution in analyzed donkey populations. Furthermore, variance component among populations within groups (P<0.05) and within populations (P<0.001) were significant.

**Table 7.** Hierarchical AMOVA analysis among the Turkish native donkey populations and 427 reference mtDNA D-loop sequences.

| **Source of variation** | **Variance component** | **Variance (%)** | **Fixation index^a^** | **p-value^b^** |
| --- | --- | --- | --- | --- |
| **Hypothesis 1: 5 different clusters** | |  |  |  |
| **Among groups** | 0.31406 | 7.95 | Φ_CT_: 0.07945 | .08211^ns^ |
| **Among populations within groups** | 0.06789 | 1.72 | Φ_SC_: 0.01866 | .0303^*^ |
| **Within populations** | 3.57081 | 90.34 | Φ_ST_: 0.09663 | .000^***^ |
| **Hypothesis 2: Turkish native donkey populations; genetic distances tree** | | | | |
| **Among groups** | 0.21621 | 7.31 | Φ_CT_: 0.07314 | 0.0303* |
| **Among populations within groups** | 0.01157 | 0.39 | Φ_SC_: 0.00422 | 0.3304^ns^ |
| **Within populations** | 2.72850 | 92.29 | Φ_ST_: 0.07705 | .000^***^ |

Φ_CT_: variation among groups divided by total variation; Φ_SC_: variation among sub-groups divided by the sum of variation among sub-groups within groups and variation within sub-groups; Φ_ST_: the sum of variation groups divided by total variation.

^b^ns= p > 0.05; *p < 0.05; ^***^p< 0.001.

Hypothesis 2; the AMOVA analyses results showed that the variation among groups, among populations within groups, and within populations were 7.31%, 0.39 % and 92.29%, respectively. The results highlight that most of the observed variance is due to differences within populations. Variance components among groups were significant (P<0.05), which is demonstrating significant geographical distribution in analyzed Turkish donkey populations by using Reynolds’ linearized genetic distances. In addition to, variance component among populations within groups non-significant but were significant within populations (P<0.001).

**4. Discussion**

As revealed in several genetic diversity studies in donkey [13,19,23,37–40], the D-loop region of maternally inherited mtDNA provides sufficient evidence to assess population genetic diversity, evolutionary relationships, and matrilineal genetic origin of a species under consideration. This study presents the substantial analysis of mtDNA diversity in Turkish native donkeys and provides information about the maternal lineage origins and genetic diversity of donkey populations, and thus insights into their genetic history.

*4.1. Phylogenetic relationships of Turkish Native Donkey Populations*

In this study, mtDNA D-loop region from 315 donkeys from 16 different Turkish native donkey populations were analyzed to clarify their phylogenetic relationship and haplogroups, and to determine mtDNA haplotypes and their maternal ancestry.

Our results showed relatively high genetic diversity within and between 16 Turkish native donkey populations from the 7 different geographical regions. Through sequence alignment, 54 distinct haplotypes were identified among Turkish donkey populations, 28 of which are unique and 26 are shared among different donkey populations. No congruence of haplogroup to a population’s geographic origin was observed. The content of A+T was the richest in the mtDNA D-loop region in Turkish donkey populations. It was in accordance with other studies, where A+T was 57.65%, whereas C+G was 42.35% [35] also matched the expected nucleotide composition of A>C>T>G with more A+T than G+C base pairs [41]. The 54 haplotypes were defined by 35 polymorphic sites (37 mutations). Of the polymorphic sites, there were 35 transitions and 2 transversions, and two sites had both a transition and transversion, suggesting a strong bias toward transitions. No insertions or deletions were observed in Turkish donkey populations. The average number of nucleotide differences (k) ranged from 4.172 to 7.333. The haplotype distribution by clusters corresponding to the Turkish native donkeys showed that two different cluster by using the maximum likelihood method (Figure 2.b). These results are found similar to Çınar Kul et al. [42] and Yalçın [43] studies.

Genetic diversity is typically measured by haplotype diversity (HD) and nucleotide diversity (πD), both of which reflect the magnitude of genetic variation at different scales [44,45]. The comparing result of HD was consistent with that of πD. We observed moderate-to-high levels of haplotype diversity in Turkish donkey populations (ranging from 0.533± 0.180 to 0.933± 0.122) and moderate nucleotide diversity (ranging from 0.01196± 0.0026 to 0.02101 ± 0.0041) indicating plentiful genetic diversity in all the Turkish donkey populations. A high level of genetic variability was observed in the AYD populations followed by KAS, MUG, ISP, KON and KUT. The overall values for haplotype and nucleotide diversity among all donkey populations was (HD: 0.763±0.023, πD: 0.01680±0.00046, respectively) similar to that estimated in other donkeys, such as the Italian donkey population donkeys (HD:0.862, πD: 0.018) [35], Anatolian donkey populations (HD: 0.756 ±0.0500, π_D_: 0.1688±0.0012) and Cypriot donkey populations (H_D_: 0.524±0.209, π_D_: 0.00176 ± 0.001) [42], Andaluza donkey populations (H_D_: 0.737; π_D_: 0.0169) [4], Somali wild ass clade (H_D_: 0.7417+0.0444) [18].

When haplotype and nucleotide diversity values for D-loop region were compared with different donkey breeds, Turkish native donkey populations found to be lesser than Ethiopian donkeys (H_D_: 0.903±0.032; π_D_: 0.020±0.003) reported by Kefena et al. [19], Balkan donkey breeds (H_D_: 0.982±0.002; π_D_: 0.017±0.009) reported by Pérez-Pardal et al. [34], Chinese donkey breeds (H_D_: 0.9055±0.017-0.9778±0.0540, π_D_: 0.02265±0.00040-0.0285±0.0160) reported by Chen et al. [17], Gan et al. [39], Lei et al. [40]; Northeast African and South American donkey populations (H_D:_ 0.737 ± 0.028- 0.910 ± 0.032; π_D_: 0.0058 ± 0.0008 - 0.0179 ± 0.0035 ) reported by Xia et al. [37], the Balkan donkey from Serbia (H_D_: 0.849±0.087; π_D_: 0.01549±0.008) reported by Stanisic et al. [15] while they were found to be higher than Spanish donkey breeds (H_D_: 0.421, π_D_: 0.0006) reported by Aranguren-Mendez et al. [4].

Pairwise F_ST_ values between Turkish native donkey populations and geographical regions were estimated. Most populations show little differentiation with F_ST_ < 0.05 (P > 0.01) or moderate but not significant differentiation (F_ST_ > 0.05, P > 0.01). However, great differentiation was found between AER, MRM, MDR, BSR and CAR region populations (KUT-KIR, KUT-CAT, KUT-KRM, KRM-AYD, KRM-KAS, KAS-CAT, KON-CAT, and KON -KRM, all F_ST_ values (F_ST_ > 0.25) (P < 0.01). Matrix of pairwise F_ST_, Reynolds linearized pair-wise matrilineal genetic distances and D_A_ genetic distances confirmed similar results between 16 Turkish native donkey populations. A Neighbor joining tree (NJT) from Reynolds’ linearized genetic distances clearly demonstrated three distinct clades in Turkish donkey populations from 7 different regions; Clade I (CAR, AER, SAR); Clade II (MDR, MRM and EAR), Clade III (BSR). NJT reconstructed using D-loop nucleotides sequence F_ST_ and D_A_ genetic distance values estimates also produced identical topology (figure not shown) to the dendrogram tree constructed using Reynolds’ linearized pair-wise genetic distance shown in Figure 3. Phylogenetic tree constructed based on Reynolds’ linearized pair-wise genetic distances indicated the same pattern of relationships. The pairwise F_ST_ values of Turkish donkey populations detected in this study was similar to the value of Mexican Creole donkeys [13], Spanish donkey breeds [4], but lower than of the Brazilian donkey population, Peruvian donkey population, Ethiopian donkey population [37], Italian donkey populations [35], Chinese donkeys [39].

The MJN analysis retrieved two major clades among the 16 different Turkish donkey populations analyzed in the present study. Two clades, separated from 5 nucleotide substitutions each, were found. These clades comprise haplotypes for which common origins are assumed since they share a characteristic pattern of mutations. As shown in the network clearly revealed two lineages and showed a star like phylogenetic pattern, in which two large haplotypes, H1 and H4, are located in the center of Clade 1 and Clade 2 lineages, respectively. The Clade 2 lineage predominated slightly (60.635%, 191/315). D- loop gene lineages in Turkish donkeys appear mixed with those of donkeys from 7 region.

*4.2. Phylogenetic relationships between Turkish native donkeys and other donkeys*

The Turkish native donkey mtDNA sequences (n=315) were compared with 427 publically available mtDNA D-loop sequences belonging to different country domestic donkey breeds, wild asses [11,15-17,23,29–35] and the horses (*Equus cabullus*, *Equus kiang)* as outgroups [16,23]*.* The aim of the compare with public mtDNA D-loop sequences was to identify the closely related populations among the Turkish native donkeys and the other worldwide regions donkey breeds.

The haplotype and nucleotide diversity values within Turkish native donkeys were (0.763±0.023) and (0.01680± 0.00046), respectively. The haplotype and nucleotide diversity values of other country donkeys ranged from (0.890±0.013), (0.01759± 0.00053) in the Chinese donkeys to (0.983±0.007), (0.02843±0.00195) in the African origin domestic donkeys, respectively. The haplotype and nucleotide diversity values of the Turkish native donkey populations was found to be lower than that of the other donkey populations (Table 5; Balkan, Chinese, Serbian, Italian etc.), indicating a relatively low level of diversity. This finding is consistent with other genetic diversity studies in different country’s donkey populations [15,34,35]. But the haplotype diversity found here was higher than that was found in a previous study in 3 different Turkish donkey populations [42], and the nucleotide diversity found here was lower than that found in that previous study. These results indicate a relatively higher level of genetic diversity in the 16 Turkish donkey populations compared with other country’s donkey populations. For example, the haplotype diversity and nucleotide diversity values of 10 Balkan donkey populations were 0.982±0.002 and 0.017±0.009 [34]. However, according to Walsh’s work on the required sample size for the diagnosis of conservation units a sample of 59 individuals is necessary to reject the hypothesis that individuals with unstamped (“hidden”) character states exist in the population size. Thus, the sample size necessary to reject a hidden state frequency of 0.05 is 56 when sampling from a finite population of 500 individuals [46]. Our genetic diversity estimation is therefore a precise reflection of Turkish native donkeys due to the large sample size used in this study.

In the study the MJN analysis identified 145 haplotypes from 742 mtDNA D-loop sequences (427 Genbank sequences and our 315 Turkish donkeys) based on 344 bp of control region sequence, and all these haplotypes grouped into five lineages. *Equus cabullus*, *Equus kiang* and Asiatic wild asses (*E. hemionus luteus, E. hemionus kulan, E. Hemionus onager*) shared (I) and (II) lineage and had their respective lineages. Lineage (I) (*Equus cabullus*) have 3 haplotypes, lineage (II) (*E. hemionus luteus, E. hemionus kulan, E. Hemionus onager, Equus kiang)* has 8 haplotypes. Four haplotypes were identified in *E. asinus somalicus* and were distributed into one lineage (III). There were 130 haplotypes in domestic donkey populations representing two lineages (IV - Clade I, V - Clade II). Out of 145 haplotypes, 54 (Clade I) are referred to Somali lineages (*Equus asinus somalicus*), whereas 75 haplotypes (Clade II) belong to Nubian lineage (*Equus africanus africanus*). The Clade I haplotypes were mainly derived from a single major haplotype (H_2_), with a simple star-like shape, whereas the genetic architecture of the Clade II haplotypes was more complicated, with more universally occurring haplotypes (e.g., H5, H37, H65). This was consistent with the much higher genetic distance and nucleotide diversity within the Clade II lineage than within the Clade I lineage, implying that the Clade II lineage involved many more individuals at the beginning of domestication compared with the Clade I lineage. In the study twenty-four haplotypes were newly discovered in Turkish native donkeys (H77-89, H92-93, H95-103). When we compare all sequences, twenty-five haplotypes were shared among the different countries donkey breeds and Turkish native donkey populations (H1-3, H5, H11-12, H14-15, H17-19, H21-22, H37, H44, H54-55, H57, H64-66, H68, H86, H90-91) [11,15–17,23,30,31,34,35]. The reduced median network showed that both lineage Somali and Nubian had genetic contribution to the Turkish native donkey evolution, as 19 haplotypes of lineage Somali (n= 191 samples) and 30 haplotypes of lineage Nubian (n=124) were identified in Turkish donkey populations. These data showed that 16 different Turkish native donkey populations and domestic other donkey populations possessed abundant mtDNA diversity and indicated two different maternal origins. Clade 1 includes the 49.59 % of sequences, whereas clade 2 includes the %48.52 % of sequences. Out of 145 haplotypes, 53 are referred to the Somali lineage (Clade I), whereas 77 haplotypes belong to Nubian lineage (Clade II). As shown in the network clearly revealed two lineages and showed a star like phylogenetic pattern, in which two large haplotypes, H2 and H5, are located in the center of Clade 1 and Clade 2 lineages, respectively. The Clade 1 lineage appeared to be predominated slightly (49.59 %, 368/742) in the domestic donkeys. The results showed that the Nubian (*Equus africanus africanus*) and Somali (*Equus asinus somali*) wild asses was the probable progenitor for ancient Turkish native donkeys and domestic donkeys. These results support previous studies on the origins of the domestic donkey [15–17,30,34,35].

In our study, to assess a hierarchical structure among Turkish native donkey populations, other country donkeys (Italian, Serbian, Chinese, etc.) and wild asses, the AMOVA analysis was performed under two different hypotheses grouping population in different clusters. In both hypotheses, the AMOVA analysis results indicate that the majority of the observed variance is due to differences among individuals within populations. The most part of the variation is observed within the individuals (90.34% Hypothesis 1 and 92.29% Hypothesis 2) whereas the differences among groups represent only the 7.95% (Hypothesis 1) and 7.31% (Hypothesis 2) of the variation, respectively. Our results are similar to a wide range of studies in mtDNA D-loop region [15,34,35,37]. In hypothesis 1; among populations within groups and among individuals within populations were statistically significant variation (P <0.001, P<0.05).

In hypothesis 2; among groups and among individuals within populations were statistically significant variation (P <0.001, P<0.05). Our AMOVA analysis and phylogenetic analysis revealed low genetic variation between the groups that were defined geographically among Turkish native donkey populations. It has been observed that there is a small difference in the donkey population of the BSR compared to the other region’s populations. In order to explain the reason for this difference, it is considered appropriate to conduct new studies with more samples in BSR.

**5. Conclusion**

In this study, it is concluded that mtDNA D-loop region sequence analysis is the most informative tool for the identification of genetic diversity, origin, and phylogeny of different donkey populations. This study demonstrated abundant mtDNA diversity existing in Turkish native donkey populations. The detection of 54 haplotypes in 315 donkeys suggests that abundant genetic diversity exists in Turkish donkey populations. We confirmed two different maternal lineages (Somali and Nubian) of domestic donkeys reported by other researchers. No obvious geographical distribution was found among Turkish native donkey populations. We present high nucleotide and haplotype diversity values, no haplotypes clearly distinct from other populations, and no clear clustering on the median joining tree in Turkish donkeys. In summary, our results suggested the relatively high genetic diversity of 16 Turkish donkey populations in mtDNA D-loop region and brought an insight in the origin of the analyzed populations. Our results clearly exclude the Asian wild donkey as progenitors of Turkish native donkey populations, and two African wild donkeys (Somali and Nubian) are the likely progenitors of Turkish native donkeys.The results presented here could be regarded as a genetic structure of the maternal lineages of Turkish native donkey populations.

**Supplementary Materials:** Some of the following tables (i.e Table S1) and some figures can be available online as supplementary materials.

**Author Contributions:** Conceptualization, M.I.S., S.A., F.Ö. and E.Ö.Ü.; sampling, F.Ö., E.Ö.Ü., E.K.G., S.G., S.K. and S.Y. ; methodology, E.Ö.Ü., F.Ö., E.K.G. and S.G.; formal analysis, E.Ö.Ü. and F.Ö., investigation, E.Ö.Ü; F.Ö., data curation, E.Ö.Ü and F.Ö.; writing—original draft preparation, E.Ö.Ü. and F.Ö.; writing—review and editing, E.Ö.Ü., E.K.G., S.G., S.K., M.I.S. and S.A.; visualization, E.Ö.Ü. and F.Ö; supervision, E.Ö.Ü. and F.Ö.; project administration, F.Ö.; funding acquisition, F.Ö. All authors have read and agreed to the published version of the manuscript.

**Funding:** This research was funded by TUBITAK (The Scientific and Technological Research Council of Turkey), grant number 215O555, project leader Fulya Özdil.

**Acknowledgments:** The authors would like to kindly thank to Selen Yatkın who provide help during the sample collection. We are also grateful to all farmers and pastoralists for allowing us to use their animal to collect blood samples for free. Many thanks to our lab team Şeref Mücahit Topaloğlu and Ayla Fidan for helping laboratory experiment. The authors want to thank four anonymous reviewers for comments on this work.

**Conflicts of Interest:** The authors declare no conflict of interest.

**References**

1. Anthony, D.W.; Bogucki, P.; Comşa, E.; Gimbutas, M.; Jovanović, B.; Mallory, J.P.; Milisaukas, S. The “Kurgan Culture,” Indo-European Origins, and the Domestication of the Horse: A Reconsideration [and Comments and Replies]. *Curr. Anthropol.* **1986**, *27*, 291–313, doi:10.1086/203441.

2. Levine, M.; Rassamakin, Y.; Kislenko, A.; Tatarintseva, N. *Late prehistoric exploitation of the Eurasian steppe (McDonald Institute Monographs)*; 1999;

3. Meadows, J.R.S.; Hanotte, O.; Drögemüller, C.; Calvo, J.; Godfrey, R.; Coltman, D.; Maddox, J.F.; Marzanov, N.; Kantanen, J.; Kijas, J.W. Globally dispersed Y chromosomal haplotypes in wild and domestic sheep. *Anim. Genet.* **2006**, *37*, 444–453, doi:10.1111/j.1365-2052.2006.01496.x.

4. Aranguren-Mendez, J.; Beja-Pereira, A.; Avellanet, R.; Dzama, K.; Jordana, J. Mitochondrial DNA variation and genetic relationships in Spanish donkey breeds (Equus asinus). *J. Anim. Breed. Genet.* **2004**, *121*, 319–330, doi:10.1111/j.1439-0388.2004.00464.x.

5. Han, L.; Yu, H.X.; Cai, D.W.; Shi, H.L.; Zhu, H.; Zhou, H. Mitochondrial DNA analysis provides new insights into the origin of the Chinese domestic goat. *Small Rumin. Res.* **2010**, *90*, 41–46, doi:10.1016/j.smallrumres.2009.12.011.

6. Cai, D.; Tang, Z.; Yu, H.; Han, L.; Ren, X.; Zhao, X.; Zhu, H.; Zhou, H. Early history of Chinese domestic sheep indicated by ancient DNA analysis of Bronze Age individuals. *J. Archaeol. Sci.* **2011**, *38*, 896–902, doi:10.1016/j.jas.2010.11.019.

7. Bruford, M.W.; Bradley, D.G.; Luikart, G. DNA markers reveal the complexity of livestock domestication. *Nat. Rev. Genet.* **2003**, *4*, 900–910, doi:10.1038/nrg1203.

8. Wilson, A.C.; Cann, R.L.; Carr, S.M.; George, M.; Gyllensten, U.B.; Helm‐Bychowski, K.M.; Higuchi, R.G.; Palumbi, S.R.; Prager, E.M.; Sage, R.D.; et al. Mitochondrial DNA and two perspectives on evolutionary genetics. *Biol. J. Linn. Soc.* **1985**, *26*, 375–400, doi:10.1111/j.1095-8312.1985.tb02048.x.

9. Gissi, C.; Reyes, A.; Pesole, G.; Saccone, C. Lineage-specific evolutionary rate in mammalian mtDNA. *Mol. Biol. Evol.* **2000**, *17*, 1022–1031, doi:10.1093/oxfordjournals.molbev.a026383.

10. Wolf, C.; Rentsch, J.; Hübner, P. PCR-RFLP analysis of mitochondrial DNA: A reliable method for species identification. *J. Agric. Food Chem.* **1999**, *47*, 1350–1355, doi:10.1021/jf9808426.

11. Xu, X.; Gullberg, A.; Arnason, U. The complete mitochondrial DNA (mtDNA) of the donkey and mtDNA comparisons among four closely related mammalian species-pairs. *J. Mol. Evol.* **1996**, *43*, 438–446, doi:10.1007/BF02337515.

12. Ivankovic, A.; Kavar, T.; Caput, P.; Mioc, B.; Pavic, V.; Dovc, P. Genetic diversity of three donkey populations in the Croatian coastal region. *Anim. Genet.* **2002**, *33*, 169–177, doi:10.1046/j.1365-2052.2002.00879.x.

13. Lopez Lopez, C.; Alonso, R.; De Aluja, A.S. Study of the genetic origin of the Mexican Creole donkey (Equus asinus) by means of the analysis of the D-loop region of mitochondrial DNA. *Trop. Anim. Health Prod.* **2005**, *37*, 173–188, doi:10.1023/B:TROP.0000049274.28640.d7.

14. Rossel, S.; Marshall, F.; Peters, J.; Pilgram, T.; Adams, M.D.; O’Connor, D. Domestication of the donkey: Timing, processes, and indicators. *Proc. Natl. Acad. Sci. U. S. A.* **2008**, *105*, 3715–3720, doi:10.1073/pnas.0709692105.

15. Stanisic, L.J.; Aleksic, J.M.; Dimitrijevic, V.; Simeunovic, P.; Glavinic, U.; Stevanovic, J.; Stanimirovic, Z. New insights into the origin and the genetic status of the Balkan donkey from Serbia. *Anim. Genet.* **2017**, *48*, 580–590, doi:10.1111/age.12589.

16. Beja-Pereira, A.; England, P.R.; Ferrand, N.; Jordan, S.; Bakhiet, A.O.; Abdalla, M.A.; Mashkour, M.; Jordana, J.; Taberlet, P.; Luikart, G. African origins of the domestic donkey. *Science (80-. ).* **2004**, *304*, 1781, doi:10.1126/science.1096008.

17. Chen, S.Y.; Zhou, F.; Xiao, H.; Sha, T.; Wu, S.F.; Zhang, Y.P. Mitochondrial DNA diversity and population structure of four Chinese donkey breeds. *Anim. Genet.* **2006**, *37*, 427–429, doi:10.1111/j.1365-2052.2006.01486.x.

18. Kimura, B.; Marshall, F.B.; Chen, S.; Rosenbom, S.; Moehlman, P.D.; Tuross, N.; Sabin, R.C.; Peters, J.; Barich, B.; Yohannes, H.; et al. Ancient DNA from Nubian and Somali wild ass provides insights into donkey ancestry and domestication. In Proceedings of the Proceedings of the Royal Society B: Biological Sciences; 2011.

19. Kefena, E.; Dessie, T.; Tegegne, A.; Beja-Pereira, A.; Yusuf Kurtu, M.; Rosenbom, S.; Han, J.L. Genetic diversity and matrilineal genetic signature of native Ethiopian donkeys (Equus asinus) inferred from mitochondrial DNA sequence polymorphism. *Livest. Sci.* **2014**, *167*, 73–79, doi:10.1016/j.livsci.2014.06.006.

20. Yılmaz, O.; Wilson, T.R. The domestic livestock resources of Turkey: Notes on donkeys. *J. Anim. Plant Sci.* **2013**, *23*, 651–656.

21. Arat, S. In vitro conservation and preliminary molecular identification of some Turkish domestic animal genetic resources (TURKAYEN-I). In Proceedings of the RBI 8th Global Conference on the Conservation of Animal Genetic Resources; 2011; pp. 51–58.

22. Sambrook, J.; Russel, D, W. Molecular Cloning, 3-Volume Set : A Laboratory Manual. *Cold Spring Harboc Lab. Press* 2001.

23. Ma, X.Y.; Ning, T.; Adeola, A.C.; Li, J.; Esmailizadeh, A.; Lichoti, J.K.; Agwanda, B.R.; Isakova, J.; Aldashev, A.A.; Wu, S.F.; et al. Potential dual expansion of domesticated donkeys revealed by worldwide analysis on mitochondrial sequences. *Zool. Res.* **2020**, *41*, 51–60, doi:10.24272/j.issn.2095-8137.2020.007.

24. Hall, T. BioEdit: An important software for molecular biology. *GERF Bull. Biosci.* **2011**, *2*, 6, doi:10.1002/prot.24632.

25. Kumar, S.; Stecher, G.; Tamura, K. MEGA7: Molecular Evolutionary Genetics Analysis Version 7.0 for Bigger Datasets. *Mol. Biol. Evol.* **2016**, *33*, 1870–1874, doi:10.1093/molbev/msw054.

26. Rozas, J.; Ferrer-Mata, A.; Sanchez-DelBarrio, J.C.; Guirao-Rico, S.; Librado, P.; Ramos-Onsins, S.E.; Sanchez-Gracia, A. DnaSP 6: DNA sequence polymorphism analysis of large data sets. *Mol. Biol. Evol.* **2017**, *34*, 3299–3302, doi:10.1093/molbev/msx248.

27. Excoffier, L.; Lischer, H.E.L. Arlequin suite ver 3.5: A new series of programs to perform population genetics analyses under Linux and Windows. *Mol. Ecol. Resour.* **2010**, *10*, 564–567, doi:10.1111/j.1755-0998.2010.02847.x.

28. Bandelt, H.J.; Forster, P.; Röhl, A. Median-joining networks for inferring intraspecific phylogenies. *Mol. Biol. Evol.* **1999**, *16*, 37–48, doi:10.1093/oxfordjournals.molbev.a026036.

29. Vilstrup, J.T.; Seguin-Orlando, A.; Stiller, M.; Ginolhac, A.; Raghavan, M.; Nielsen, S.C.A.; Weinstock, J.; Froese, D.; Vasiliev, S.K.; Ovodov, N.D.; et al. Mitochondrial phylogenomics of modern and ancient equids. *PLoS One* **2013**, *8*, e55950, doi:10.1371/journal.pone.0055950.

30. Sun, Y.; Jiang, Q.; Yang, C.; Wang, X.; Tian, F.; Wang, Y.; Ma, Y.; Ju, Z.; Huang, J.; Zhou, X.; et al. Characterization of complete mitochondrial genome of Dezhou donkey (Equus asinus) and evolutionary analysis. *Curr. Genet.* **2016**, *62*, 383–390, doi:10.1007/s00294-015-0531-9.

31. Schubert, M.; Mashkour, M.; Gaunitz, C.; Fages, A.; Seguin-Orlando, A.; Sheikhi, S.; Alfarhan, A.H.; Alquraishi, S.A.; Al-Rasheid, K.A.S.; Chuang, R.; et al. Zonkey: A simple, accurate and sensitive pipeline to genetically identify equine F1-hybrids in archaeological assemblages. *J. Archaeol. Sci.* **2017**, *78*, 147–157, doi:10.1016/j.jas.2016.12.005.

32. Luo, Y.; Chen, Y.; Liu, F.; Jiang, C.; Gao, Y. Mitochondrial genome sequence of the Tibetan wild ass (Equus kiang). *Mitochondrial DNA* **2011**, *22*, 6–8, doi:10.3109/19401736.2011.588221.

33. Jónsson, H.; Schubert, M.; Seguin-Orlando, A.; Ginolhac, A.; Petersen, L.; Fumagalli, M.; Albrechtsen, A.; Petersen, B.; Korneliussen, T.S.; Vilstrup, J.T.; et al. Speciation with gene flow in equids despite extensive chromosomal plasticity. *Proc. Natl. Acad. Sci. U. S. A.* **2014**, *111*, 18655–18660, doi:10.1073/pnas.1412627111.

34. Pérez-Pardal, L.; Grizelj, J.; Traoré, A.; Cubric-Curik, V.; Arsenos, G.; Dovenski, T.; Marković, B.; Fernández, I.; Cuervo, M.; Álvarez, I.; et al. Lack of mitochondrial DNA structure in Balkan donkey is consistent with a quick spread of the species after domestication. *Anim. Genet.* **2014**, doi:10.1111/age.12086.

35. Cozzi, M.C.; Valiati, P.; Cherchi, R.; Gorla, E.; Prinsen, R.T.M.M.; Longeri, M.; Bagnato, A.; Strillacci, M.G. Mitochondrial DNA genetic diversity in six Italian donkey breeds (Equus asinus). *Mitochondrial DNA Part A DNA Mapping, Seq. Anal.* **2018**, *29*, 409–418, doi:10.1080/24701394.2017.1292505.

36. Achilli, A.; Olivieri, A.; Soares, P.; Lancioni, H.; Kashani, B.H.; Perego, U.A.; Nergadze, S.G.; Carossa, V.; Santagostino, M.; Capomaccio, S.; et al. Mitochondrial genomes from modern horses reveal the major haplogroups that underwent domestication. *Proc. Natl. Acad. Sci. U. S. A.* **2012**, *109*, 2449–2454, doi:10.1073/pnas.1111637109.

37. Xia, X.; Yu, J.; Zhao, X.; Yao, Y.; Zeng, L.; Ahmed, Z.; Shen, S.; Dang, R.; Lei, C. Genetic diversity and maternal origin of Northeast African and South American donkey populations. *Anim. Genet.* **2019**, *50*, 266–270, doi:10.1111/age.12774.

38. Han, L.; Zhu, S.; Ning, C.; Cai, D.; Wang, K.; Chen, Q.; Hu, S.; Yang, J.; Shao, J.; Zhu, H.; et al. Ancient DNA provides new insight into the maternal lineages and domestication of Chinese donkeys. *BMC Evol. Biol.* **2014**, *14*, 246, doi:10.1186/s12862-014-0246-4.

39. Gan, Q.F.; Lin, S.X.; Liang, X.W.; Meng, H.L.; Qiao, H.; Du, Y.F.; Ke, F.R.; Zheng, H.K. African maternal origin and genetic diversity of five chinese domestic donkeys breeds. *J. Anim. Vet. Adv.* **2011**, *10*, 3090–3094, doi:10.3923/javaa.2011.3090.3094.

40. Lei, C.Z.; Ge, Q.L.; Zhang, H.C.; Liu, R.Y.; Zhang, W.; Jiang, Y.Q.; Dang, R.H.; Zheng, H.L.; Hou, W.T.; Chen, H. African maternal origin and genetic diversity of Chinese domestic donkeys. *Asian-Australasian J. Anim. Sci.* **2007**, *20*, 645–652, doi:10.5713/ajas.2007.645.

41. İBİŞ, O. Mitogenome characterization of Turkish Anatolian Donkey (Equus asinus) and Its phylogenetic relationships. *Türkiye Tarımsal Araştırmalar Derg.* **2019**, *6*, 257–267, doi:10.19159/tutad.569435.

42. Cinar Kul, B.; Bilgen, N.; Akyuz, B.; Ertugrul, O. Molecular phylogeny of Anatolian and Cypriot donkey populations based on mitochondrial DNA and Y-chromosomal STRs. *Ankara Univ. Vet. Fak. Derg.* **2016**, *63*, 143–149, doi:10.1501/vetfak_0000002722.

43. Yalçın, E. Kırklareli ili eşek çiftliği populasyonlarının çeşitli vücut ölçülerine göre morfometrik karakterizasyonu ve mtDNA polimorfizmi yoluyla genetik çeşitliliğinin incelenmesi, 2016, Namık Kemal University Graduate School of Natural and Applied Sciences Master of Science Thesis. 90 pp.

44. Tajima, F. Evolutionary relationship of DNA sequences in finite populations. *Genetics* **1983**, *105*, 437–460.

45. Nei, M. Phylogenetic analysis in molecular evolutionary genetics. *Annu. Rev. Genet.* **1996**, *30*, 371–403, doi:10.1146/annurev.genet.30.1.371.

46. Walsh, P.D. Sample size for the diagnosis of conservation units. *Conserv. Biol.* **2000**, *14*, 1533–1537, doi:10.1046/j.1523-1739.2000.98149.x.
