# Supplementary material for: Phylogenetic Relationships of Turkish Indigenous Donkey Populations Determined by Mitochondrial DNA D-loop Region
Source: Animals (Basel). 2020 Oct 27;10(11):1970. doi: 10.3390/ani10111970 (PMC7692571; doi:10.3390/ani10111970)
Supplement: Supplementary file 1 [file animals-10-01970-s001.zip › Supp_Mat_Table_S2_S4_Fig1.docx]

**Supplementary Table S2:** Matrix of pairwise fixation indexes (F_ST_) calculated for the 16 Turkish native donkey populations.

|  | **KIR** | **CAT** | **MAL** | **MER** | **TOK** | **KAS** | **KUT** | **MUG** | **AYD** | **KON** | **ISP** | **KRM** | **ANT** | **KAR** | **MRD** | **SAN** |
| --- | --- | --- | --- | --- | --- | --- | --- | --- | --- | --- | --- | --- | --- | --- | --- | --- |
| **KIR** | - | -0.02866 | -0.06373 | -0.02520 | -0.02032 | 0.22264 | 0.25303 | 0.02326 | 0.18195 | 0.22110 | -0.01517 | -0.00877 | -0.02395 | -0.02886 | 0.04968 | 0.03608 |
| **CAT** |  | - | -0.05104 | -0.01319 | 0.01983 | 0.28436 | 0.31643 | 0.07714 | 0.24229 | 0.28478 | 0.01608 | -0.02891 | 0.01336 | -0.01598 | 0.09119 | 0.07906 |
| **MAL** |  |  | - | -0.06174 | -0.07600 | 0.14815 | 0.18224 | -0.05329 | 0.11138 | 0.15188 | -0.06730 | -0.03143 | -0.07586 | -0.06465 | -0.00581 | -0.01909 |
| **MER** |  |  |  | - | -0.03023 | 0.19163 | 0.21907 | -0.01055 | 0.15843 | 0.19996 | -0.02885 | 0.00257 | -0.02830 | -0.02558 | 0.03997 | 0.02685 |
| **TOK** |  |  |  |  | - | 0.04640 | 0.07687 | -0.11835 | 0.02376 | 0.04705 | -0.07984 | 0.04300 | -0.08850 | -0.03652 | -0.04541 | -0.06125 |
| **KAS** |  |  |  |  |  | - | -0.05773 | -0.05946 | -0.03652 | -0.03929 | 0.04428 | 0.30177 | 0.07146 | 0.18566 | 0.03271 | 0.03855 |
| **KUT** |  |  |  |  |  |  | - | -0.05753 | -0.04453 | -0.04312 | 0.06021 | 0.34463 | 0.10193 | 0.20780 | 0.04229 | 0.06238 |
| **MUG** |  |  |  |  |  |  |  | - | -0.06567 | -0.04353 | -0.11043 | 0.10654 | -0.09026 | -0.01150 | -0.08453 | -0.08411 |
| **AYD** |  |  |  |  |  |  |  |  | - | -0.04880 | 0.04003 | 0.26814 | 0.04258 | 0.14627 | 0.00908 | 0.01653 |
| **KON** |  |  |  |  |  |  |  |  |  | - | 0.07378 | 0.31352 | 0.07208 | 0.18433 | 0.02958 | 0.03529 |
| **ISP** |  |  |  |  |  |  |  |  |  |  | - | 0.03288 | -0.07191 | -0.03540 | -0.03734 | -0.03788 |
| **KRM** |  |  |  |  |  |  |  |  |  |  |  | - | 0.03579 | 0.00470 | 0.11355 | 0.09992 |
| **ANT** |  |  |  |  |  |  |  |  |  |  |  |  | - | -0.03800 | -0.03641 | -0.04750 |
| **KAR** |  |  |  |  |  |  |  |  |  |  |  |  |  | - | 0.02630 | 0.01638 |
| **MRD** |  |  |  |  |  |  |  |  |  |  |  |  |  |  | - | -0.02382 |
| **SAN** |  |  |  |  |  |  |  |  |  |  |  |  |  |  |  | - |

**Supplementary Table S3:** Reynolds’ linearized pair-wise matrilineal genetic distance between 7 region donkey populations.

|  | **MRM** | **BSR** | **AER** | **MDR** | **CAR** | **EAR** | **SAR** |
| --- | --- | --- | --- | --- | --- | --- | --- |
| **MRM** | - |  |  |  |  |  |  |
| **BSR** | 0.0245 | - |  |  |  |  |  |
| **AER** | 0.2364 | 0.0796 | - |  |  |  |  |
| **MDR** | 0.0000 | 0.0069 | 0.1905 | - |  |  |  |
| **CAR** | 0.2907 | 0.1083 | 0.0000 | 0.2395 | - |  |  |
| **EAR** | 0.0000 | 0.0000 | 0.1583 | 0.0000 | 0.2037 | - |  |
| **SAR** | 0.07097 | 0.0004 | 0.0295 | 0.0492 | 0.0383 | 0.0266 | - |

**Supplemental Table S4.** GenBank accession numbers of the 217 donkey mtDNA D-loop sequences used in the present study.

| **Accession number** | **N** | **Breed** | **Reference** |
| --- | --- | --- | --- |
| **X97337, MK896308** | 2 | Ref. mtDNA samples | Xu et al., 1996; Ma et al., 2020 |
| **JN398377** | 1 | *Equus caballus* (horse) | Achilli et al., 2012 |
| **AY569462 - 551** | 85 | \| African origin domestic donkey \| \| --- \| | Beja-Pereira et al., 2004 |
| **JX312728, JX312730** | 2 | *Equus hemionus kulan (*Transcaspian wild ass*),*  *Equus hemionus onager* (Persian wild ass) | Vilstrup et al., 2013 |
| **JX472930-91** | 62 | Balkan donkeys | Perez-Pardal et al., 2014 |
| **NC_001788** | 1 | *Equus asinus* | Xu et al., 1996 |
| **NC_016061** | 1 | *Equus hemionus* | Lu et al., 2011 |
| **DQ448878–9023** | 146 | Chinese donkey | Chen et al., 2006 |
| **NC_018782, NC_020433** | 2 | [*Equus hemionus kulan*](https://www.ncbi.nlm.nih.gov/Taxonomy/Browser/wwwtax.cgi?id=73334)*; Equus kiang* | Vilstrup et al., 2013 |
| **KR081377-95, KR081392, KR081391,** | 21 | Serbian donkey populations | Stanisic et al., 2017 |
| **MK619357–411; MK896291-308** | 73 | China, Tadzhikistan, Kyrgyzstan, Iran, Kenya, Mongolia | Ma et al., 2019 |
| **KM881681** | 1 | *Equus asinus somalicus* | Jonsson et al., 2014 |
| **KT182635** | 1 | Dezhou donkey | Sun et al., 2015 |
| **KX669267** | 1 | *Equus asinus africanus* | Schubert et al., 2017 |
| **KX622700-727** | 28 | Italian donkey breeds | Cozzi et al., 2018 |
| **Turkish native donkeys** | 315 | 16 different populations | Present study |
| **Total** | 743 |  |  |

| **Supplementary Figure 1.** Mitochondrial D-loop sequence variations detected in 145 haplotypes of 315 Turkish native donkeys and 429 429 reference mtDNA D-loop sequences. N referred to the number of samples.  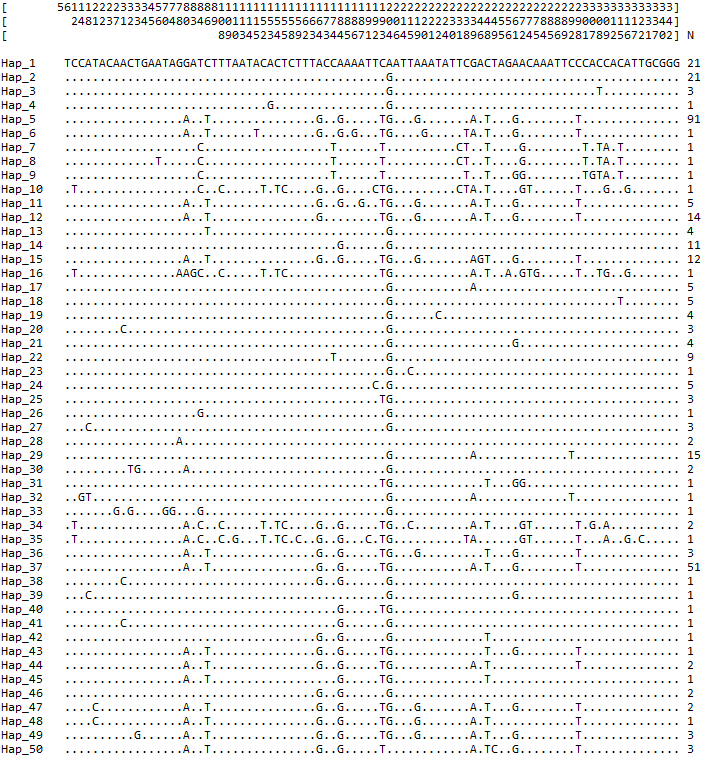 |
| --- |
| **Supplementary Figure 1.** Mitochondrial D-loop sequence variations detected in 145 haplotypes of 315 Turkish native donkeys and 429 429 reference mtDNA D-loop sequences. N referred to the number of samples.  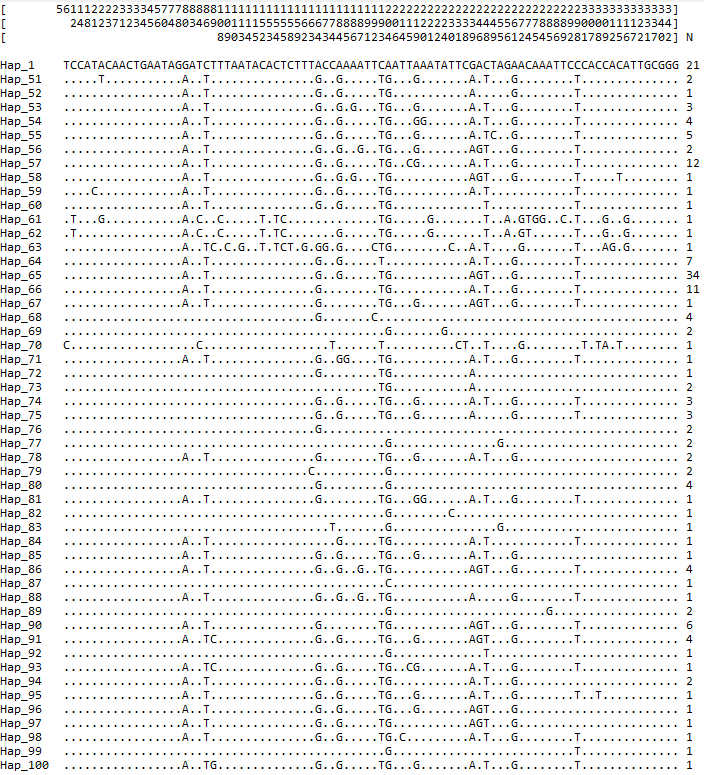 |
| **Supplementary Figure 1.** Mitochondrial D-loop sequence variations detected in 145 haplotypes of 315 Turkish native donkeys and 429 429 reference mtDNA D-loop sequences. N referred to the number of samples.  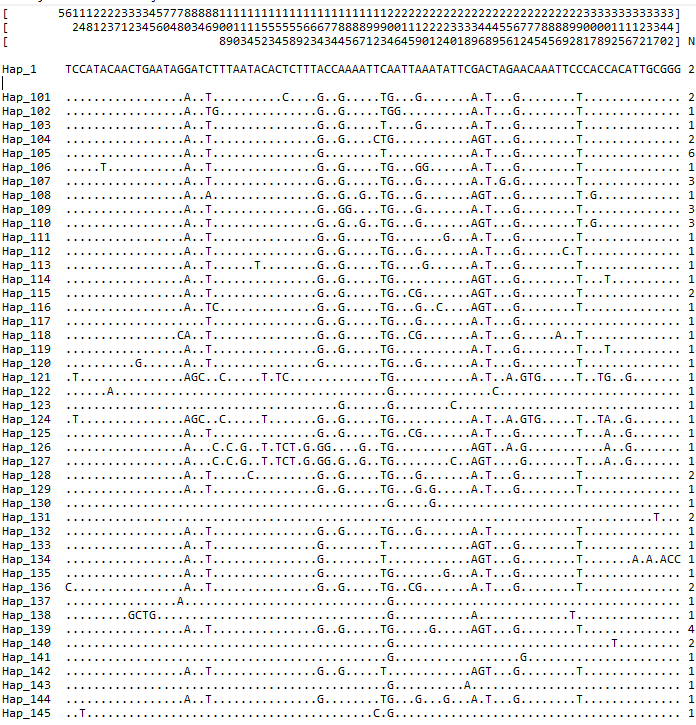 |
